# From Device-Level Implementation to In-Sensor Computing in Memristive-Device-Based Biosensors: A Review

**DOI:** 10.3390/bios16080444

**Published:** 2026-08-16

**Authors:** Hyunwook Ryu, Won-Chul Lee, Jongwon Lee

**Affiliations:** 1 Department of Electronics Engineering, Chungnam National University, Daejeon 34134, Republic of Korea; 2 Division of Nano Convergence Technology Development, National NanoFab Center (NNFC), Daejeon 34141, Republic of Korea; 3 Department of Semiconductor Convergence, Chungnam National University, Daejeon 34134, Republic of Korea

**Keywords:** memristive device, biosensor, in-sensor computing, edge AI

## Abstract

Memristive devices have attracted considerable attention as promising candidates for overcoming the energy and data-transfer limitations of conventional computing architectures. In particular, their integration with biosensors offers a pathway toward compact and energy-efficient diagnostic systems. This review examines the development of memristive-device-based biosensors from device-level transduction to system-level integration. At the device level, sensing strategies have evolved from direct sensing toward indirect sensing architectures, improving stability and reusability. At the system level, conventional off-chip implementations have progressively shifted toward fully integrated on-chip implementations. Furthermore, this review highlights the emerging paradigm of in-sensor computing, in which sensing, memory, and computation are co-located within a single physical platform. This approach enables reduced data movement and supports energy-efficient operation for point-of-care applications. Finally, key challenges—including CMOS compatibility, device variability, and reliable multi-threshold sensing operation—are discussed as critical factors for the practical realization of memristive-device-based electrochemical biosensing systems.

## 1. Introduction

Conventional electrochemical biosensing systems typically rely on a spatially separated architecture where the transducer and the central processing unit are physically decoupled. Following the standard von Neumann architecture, raw analog signals acquired at the sensor edge must be transferred to external processors for digitization and analysis. This structural separation introduces inherent data transfer bottlenecks and latency, which contribute significantly to power consumption and operational delays in conventional hardware architectures [1,2]. As the demand for continuous biological monitoring and point-of-care (POC) testing increases, the continuous transmission of unclassified raw data poses a challenge to the energy efficiency of conventional silicon-based computing technologies, which are facing increasing scaling challenges [3,4,5,6].

To address these architectural limitations, in-sensor computing has emerged as a structural alternative that integrates computational capabilities directly at or immediately adjacent to the sensing interface [7]. Memristive devices provide a hardware foundation for this integration by inherently resolving the physical separation between memory and logic elements [8,9,10]. Since their theoretical conceptualization as a fundamental circuit element [11] and subsequent experimental realization in thin-film oxides [12], memristive devices have been widely recognized for their non-volatile operation, low-voltage switching, and nanoscale form factor.

This review provides an overview of the device-level innovations and system-level architectures, and the emerging in-sensor computing paradigms associated with memristor-based biosensing architectures. At the device level, the implementation of memristive devices into biosensing platforms has systematically evolved to balance high transduction sensitivity with long-term operational reliability. Initial configurations predominantly utilized direct exposure strategies, where the active memristive layer was placed in physical contact with biological solutions to exploit ion-sensitive switching behaviors. However, prolonged exposure to complex electrolyte environments frequently induces chemical degradation and structural instability in the active materials. Consequently, recent developments have shifted toward indirect sensing configurations, where the core memristive component is physically decoupled from the liquid medium but remains electrically interconnected to process the primary transducer output [13,14]. Building upon this, the current trajectory is directed toward complete, fully decoupled in-sensor computing nodes that perform deterministic threshold sensing-based decision logic using replaceable sensing electrodes, thereby entirely preventing material degradation while retaining data-stationary processing capabilities at the transducer level [15].

Following device-level advances in transduction and stability, memristive-device-based biosensing platforms have increasingly moved toward system-level integration of readout and signal-processing architectures. Off-chip implementations have combined memristive biosensor arrays or electrochemical sensors with PCB-based, smartphone-based, or microfluidic readout platforms to improve automated measurement, portability, and practical point-of-care operation [16,17,18]. In parallel, on-chip neuromorphic biosensor systems have integrated sensing, memory, and classification stages on a single substrate, enabling localized learning and classification without relying entirely on external software [19]. However, these system-level platforms still retain architectural limitations, including dependence on external processors, physically separated sensing and computing modules, or continuous data transfer between the sensing interface and the processing unit.

Building on this system-level transition, in-sensor computing has emerged as a more integrated paradigm in which sensing, memory, and primary computation are co-located at or near the transducer [7]. In memristive-device-based electrochemical biosensors, this concept is represented by threshold-sensing transducers that convert analyte-dependent signals into non-volatile resistance states, enabling local decision logic without fully relying on sequential digital conversion or external processing [15]. Beyond electrochemical sensing, related in-sensor computing platforms have further demonstrated that local feature extraction and neuromorphic signal processing can be implemented directly at the sensing edge in optical, tactile, wearable, and photonic systems [20,21,22,23].

The organization of this review is as follows. Section 2 defines the foundational terminology and scope of this review. Section 3, Section 4 and Section 5 discuss device-level implementations, system-level implementations, and in-sensor computing trends in memristive-device-based biosensing platforms, respectively. Section 6 outlines emerging technological trends and future perspectives, discussing diverse technical expansions. Section 7 discusses the remaining practical bottlenecks. Finally, Section 8 concludes the review.

## 2. Concepts and Scope of the Review

To establish a clear conceptual foundation for this review, the fundamental terminology is defined in accordance with established standards. The International Union of Pure and Applied Chemistry (IUPAC) defines a biosensor as a device that uses specific biochemical reactions mediated by isolated enzymes, immunosystems, tissues, organelles, or whole cells to detect chemical compounds, usually through electrical, thermal, or optical signals [24]. Based on this definition, the term biosensor is used in this review for platforms involving biological or biochemical target recognition and detection. At the same time, this review traces the architectural transition from memristive-device-based biosensing toward in-sensor computing. Therefore, selected sensor and in-sensor-computing platforms beyond conventional biochemical detection are also discussed to identify transferable integration strategies for co-locating sensing, memory, and computation across device, array, and system levels.

Building upon this framework, the scope of this review adopts the inclusive term memristive devices for the corresponding signal processing components. As research in the field of memristors has rapidly diversified across a vast array of materials and physical architectures, establishing precise terminology is essential to prevent conceptual confusion. While classical definitions often restrict memristors to two-terminal passive elements, in the context of integrating sensing and computation, memristive devices are defined functionally by their ability to exhibit non-volatile hysteresis, emulate synaptic plasticity and retain memory states [25,26]. Therefore, this review encompasses not only conventional two-terminal structures but also advanced three-terminal configurations that exhibit hysteretic or memory-related sensing behavior, such as selected nanowire field-effect transistor (FET) platforms [27,28,29] and hybrid one-transistor/one-resistor (1T1R) electronic synapses [30]. Conventional nanowire FET biosensors are also discussed as field-effect sensing benchmarks when they provide relevant insights into transduction sensitivity, surface functionalization, or device stability [31,32,33,34]. By adopting this functional classification, we aim to comprehensively cover the device-level innovations that enable localized data processing.

Within the localized processing frameworks evaluated in this review, threshold-sensing (TS) operation is defined as an architectural bio-transduction paradigm in which a memristive-device-based transducer performs built-in decision logic at the sensing interface. This term is distinct from threshold-switching behavior, which refers to an intrinsic device operation in which a memristive device transitions from a high resistance state (HRS) to a low resistance state (LRS). This behavior occurs when the applied voltage exceeds a characteristic threshold, while a return to the HRS occurs if the applied voltage is reduced below a relevant threshold.

In TS operation, the analyte-dependent output signal generated by the sensing circuit determines whether the memristive element changes state once a predefined threshold condition is satisfied. Extending this concept, multi-threshold sensing operation refers to a circuit-level threshold-sensing capability in which multiple programmable threshold values are established [15].

## 3. Device Level Implementations

### 3.1. Direct Sensing via Electrostatic Field-Effect Modulations

Early research on memristor-based biosensors predominantly concentrated on achieving high sensitivity through direct interaction between the analyte and the electrode interface of the device. By directly exposing nanowires or metal oxide active layers, minute charge variations were instantaneously converted into resistance changes, thereby maximizing transduction efficiency. Nanowire-based FET biosensors first provided an important field-effect sensing benchmark for highly sensitive, label-free biomolecular detection [31,32,33,34]. Building on this field-effect sensing framework, selected silicon nanowire (SiNW) platforms exploited analyte-induced hysteresis and memory-related electrical behavior for memristive biosensing [27,28,29].

As highlighted in recent reviews [31], the high surface-to-volume ratio of SiNWs allows subtle surface charge variations to effectively modulate the depletion region and channel conductance. To enhance sensing performance in a related nanowire FET platform, the InP nanowire surface was functionalized with ethanolamine and polyethylene glycol (PEG), increasing receptor density while improving surface uniformity [33]. This approach reduces steric hindrance and facilitates effective Debye-length matching, thereby promoting robust analyte binding. Crucially for biological applications, this mechanism enables real-time, label-free detection, as the nanoscale dimensions of the wire are highly compatible with the size of biological targets. Upon binding of charged biomolecules, the carrier concentration within the nanowire channel is modulated, leading to measurable changes in conductance. Based on this mechanism, researchers have achieved ultra-sensitive detection of various targets, translating molecular interactions directly into electrical signals. In detail, protein biomarkers have been detected at concentrations as low as 5.7 fM [33], and viral targets such as the Ebola virus have been successfully identified at concentrations as low as 6 fM [29]. These advancements established a foundation for femtomolar-level, high-sensitivity biosensing using SiNW-based FET platforms. However, this FET-based sensing mechanism inherently requires continuous biasing, resulting in constant power consumption. Moreover, operation in electrolyte environments makes the sensor susceptible to ionic noise and measurement instability, limiting practical reliability in biological conditions.

Sun et al. [32] demonstrated the high sensitivity achievable with FET-based SiNW sensors for clinical diagnostics. To meet the demand for early diagnosis of chronic hepatitis B, they developed a p-type SiNW array FET capable of joint detection of Hepatitis B Virus (HBV) and Alpha-fetoprotein (AFP). By leveraging the large specific surface area of SiNWs to maximize conductance changes upon trace target binding and utilizing a dense nanowire array structure that amplifies the signal-to-noise ratio, they achieved a limit of detection (LOD) as low as 0.1 fM for HBV-DNA and 0.1 fg/mL for AFP. This result suggests that the combination of the large specific surface area and a uniform array architecture provides a label-free, robust foundation for highly sensitive clinical diagnostics. However, despite this ultra-low LOD, the FET-based sensing mechanism inherently relies on real-time conductance measurements, which necessitate continuous biasing. This leads to persistent power consumption and increases susceptibility to measurement instability. Such fundamental operational constraints pose a critical challenge for the deployment of these sensors in low-power, continuous monitoring applications.

**Figure 1:**
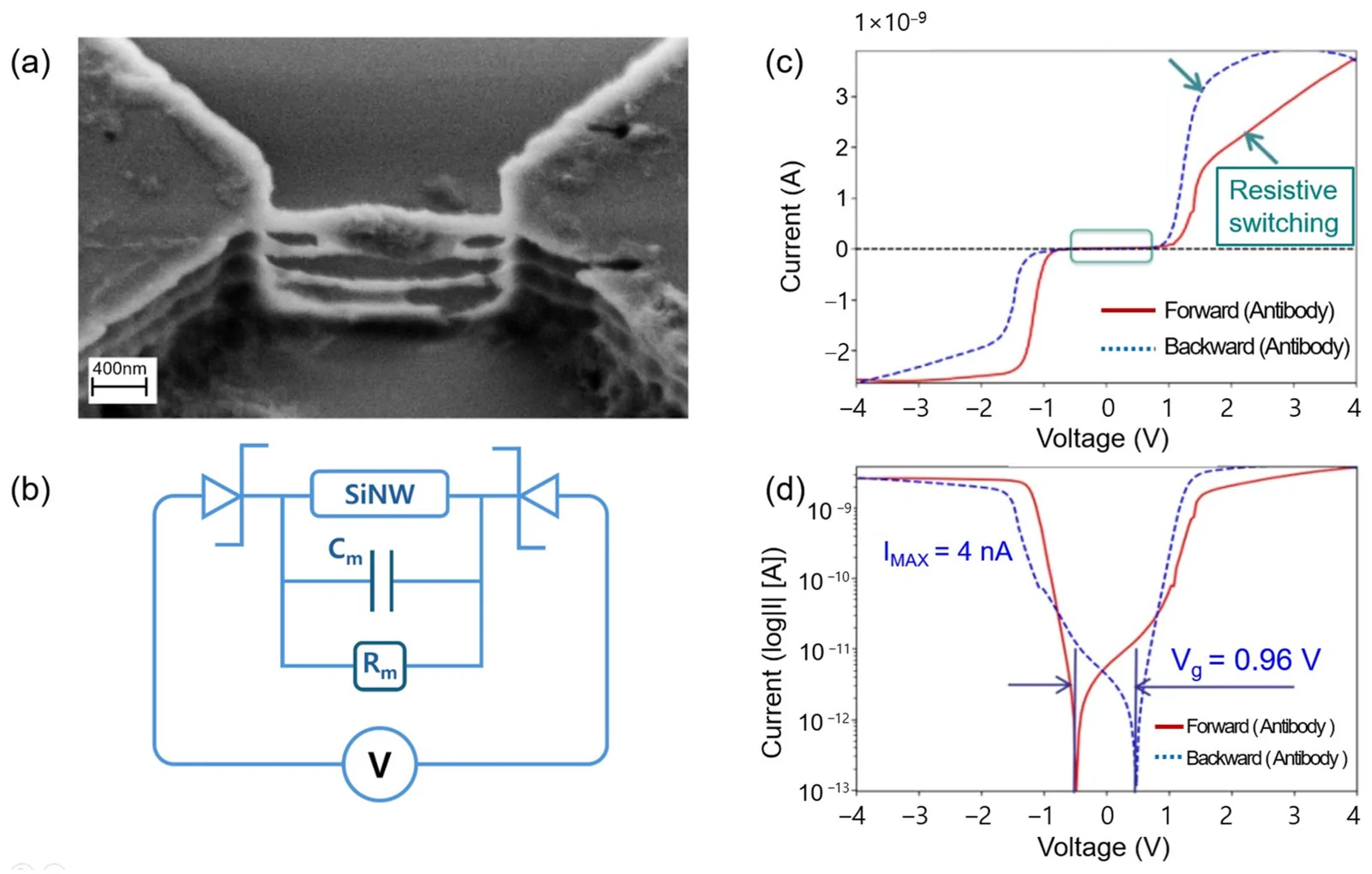
Memristive SiNW biosensor architecture and its threshold-switching mechanism. (**a**) Scanning electron microscopy (SEM) image of the suspended SiNW device. (**b**) Equivalent circuit model demonstrating the parallel combination of the nanowire’s intrinsic resistance and capacitance, flanked by Schottky barriers. (**c**) I-V characteristics exhibiting pronounced hysteresis during bidirectional voltage sweeps. (**d**) Logarithmic scale I-V curve defining the distinct voltage gap (V_g_ = 0.96 V) generated by the memristive capacitive effect. Adapted with permission from [27].

In conventional SiNW FETs, hysteresis observed during voltage sweeps has long been regarded as an undesirable effect arising from charge trapping. However, Chen and Carrara et al. [27] reinterpreted this behavior by defining the voltage gap (V_g_), induced by biomolecular binding, as a key sensing metric, thereby establishing the basis for memristive sensing. In their device, Schottky barriers at the NiSi/SiNW interfaces introduce trap states that enable charge capture and release, resulting in controllable Current-Voltage (I-V) hysteresis. When target molecules bind to the surface, their intrinsic charges form a parallel combination of capacitance and resistance (C_bio_//R_bio_), which alters the minimum current position, generating a measurable V_g_. By selectively localizing analyte molecules on the SiNW surface, a V_g_ of 0.96 V and stable resistive switching were achieved, demonstrating the feasibility of memristive-device-based sensors capable of simultaneous sensing and memory functions (Figure 1). Despite the functionality, the proposed scheme is vulnerable to spatial contamination, according to the authors. In contrast, when biomolecules are non-uniformly distributed over both the SiNW and NiSi surfaces, the resulting molecular layer can short the Schottky-diode contacts at the Si–NiSi contact regions, thereby eliminating the rectification effect and causing the loss of resistive-switching behavior. This constraint accelerates irreversible chemical degradation, thereby severely limiting device reusability and operational lifetime.

**Figure 2:**
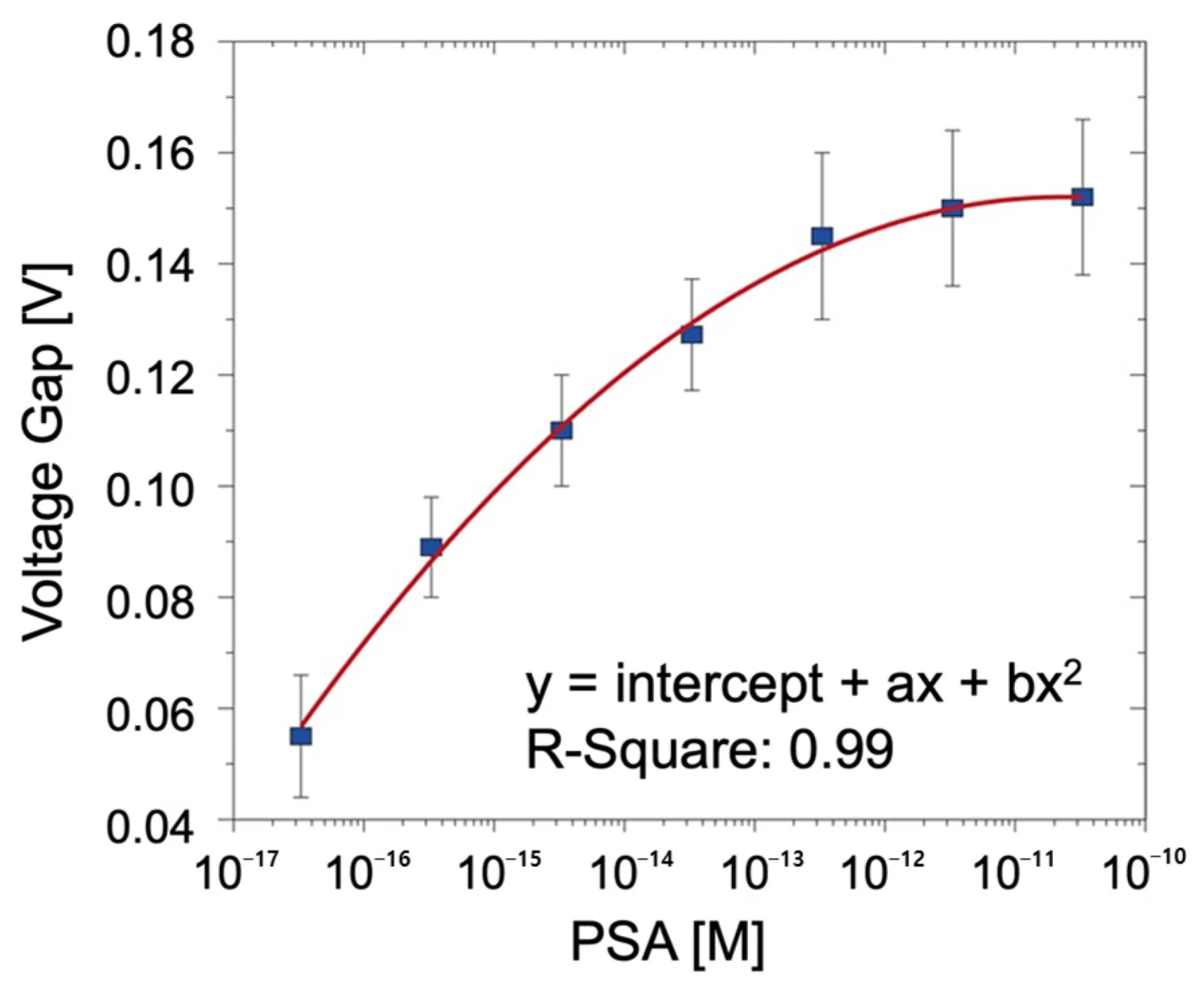
Dose–response curve of the memristive aptasensor for Prostate-Specific Antigen (PSA) detection. The plot shows the proportional expansion of the voltage gap (V_g_) with increasing PSA concentration, demonstrating an ultra-sensitive limit of detection of 23 aM [28].

Building upon Carrara’s architectural foundation, Tzouvadaki et al. [28] reported a memristive aptasensor based on a field-effect architecture designed for the label-free detection of Prostate Specific Antigen (PSA) as shown in Figure 2. The binding of PSA to DNA aptamers on the SiNW surface contributes extra charges that generate an “all-around biogate effect,” effectively modulates carrier distribution solely by the analyte, eliminating the need for an external gate voltage. The analyte-induced electric field alters the carrier distribution within the nanowire, shifting the position of the minimum current depending on the voltage sweep direction and thereby expanding the voltage gap (V_g_). Experimentally, this approach provided a highly reliable sensing signal with V_g_ increasing from a baseline of 0.027 V to 0.152 V at 33 pM of PSA. Ultimately, the sensor achieved an ultra-LOD of 23 aM (2.3 × 10^−17^ M), surpassing the ~0.1 fM range of conventional FET-based sensors. However, despite the exceptional LOD enabled by the all-around biogate effect, the maximum V_g_ margin is significantly reduced (0.152 V) compared to earlier antibody-based designs (0.96 V). This narrow margin for memristive operation increases susceptibility to noise intrinsic in biological fluids, thereby complicating reliable signal discrimination and processing.

To mitigate these device-level constraints and secure larger operational margins, neuromorphic hardware integration has progressively expanded from single passive channels toward multi-terminal gated configurations. Milo et al. [30] demonstrated that co-integrating a three-terminal active modulation framework within a hybrid 1T1R synaptic architecture provides deterministic and robust control over structural plasticity. In this configuration, the transistor functions strictly as a selector to isolate the cell and modulate compliance current, while the resistive switching occurs exclusively within the two-terminal memory element. By decoupling the signal-biasing node from the non-volatile memory element, this multi-terminal layout effectively immunizes the core computing channel against the stochastic noise and operational instabilities inherent in standalone two-terminal or unbuffered nanowire pathways.

### 3.2. Direct Sensing via Electrochemical Filamentary and Interfacial Redox Reactions

While field-effect-based platforms predominantly leverage surface electrostatic gating, the studies discussed in this section [35,36,37] exploit the intrinsic electronic switching behavior of multi-state channels. Predominantly utilizing BaTiO_x_, NbTiO_x_, and GdO_x_, these studies quantified the impact of analyte interactions on internal state variables, oxygen vacancy-driven filament formation and interfacial Schottky barrier modulation, marking a transition toward device-centric sensing mechanisms.

**Figure 3:**
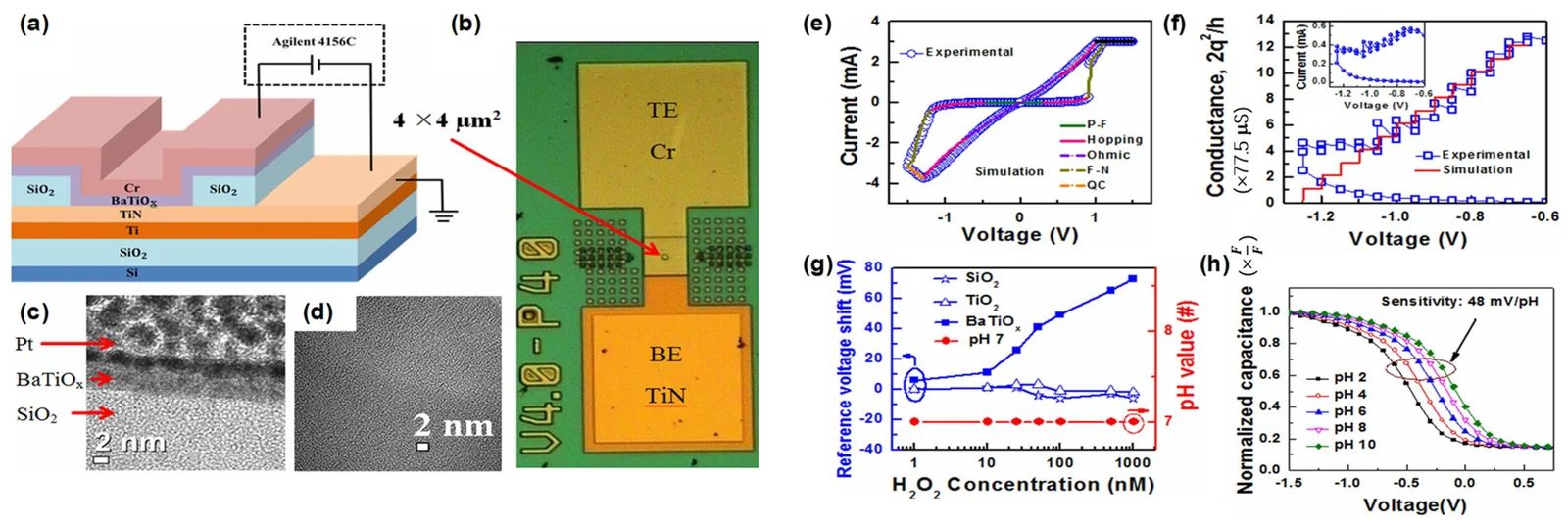
Device architecture and sensing performance of the BaTiO_x_-based memristive sensor. The structural panels (**a**–**d**) show the schematic and cross-sectional TEM images of the TiN/BaTiO_x_/Cr device. The electrical characterization panels (**e**–**h**) highlight the discrete quantum-conductance steps and the pH- and H_2_O_2_-sensing responses of the BaTiO_x_ membrane [35].

Chakrabarti et al. [35] developed a device comprising an amorphous BaTiO_x_ active layer (Figure 3a–d) for detecting hydrogen peroxide (H_2_O_2_), a key reactive oxygen species (ROS) associated with glucose metabolism and cancer-related oxidative stress. By evaluating the film in an Electrolyte–Insulator–Semiconductor structure, they successfully detected H_2_O_2_ down to 1 nM with a sensitivity of 48 mV/pH (Figure 3g,h). H_2_O_2_ sensing was used to probe the redox behavior and oxidation-state changes in Ba-related species in the BaTiO_x_ membrane. However, the resistive switching and multi-level operation in the fabricated TiN/BaTiO_x_/Cr memory stack are more appropriately described in terms of oxygen-ion/vacancy migration, conductive-filament formation, and gradual filament dissolution during the RESET process. The device exhibited resistive switching with an excellent on/off ratio of 2000 (Figure 3e). As the conductive filament diameter approaches the nanoscale, the device exhibits quantized conductance in integer multiples of G_0_ = 2e^2^/h (approx. 77.5 µS)and multi-level operation (Figure 3f), validating a robust platform for memristor-based biosensing. However, several limitations may remain. Continuous exposure to highly reactive H_2_O_2_ solutions is likely to induce material degradation, yet systematic reliability analysis under prolonged exposure is not provided. In addition, the material stack, particularly BaTiO_x_ and Cr, does not fully conform to standard Complementary Metal-Oxide-Semiconductor (CMOS) fabrication. This raises concerns regarding large-scale integration and manufacturability for practical applications.

**Figure 4:**
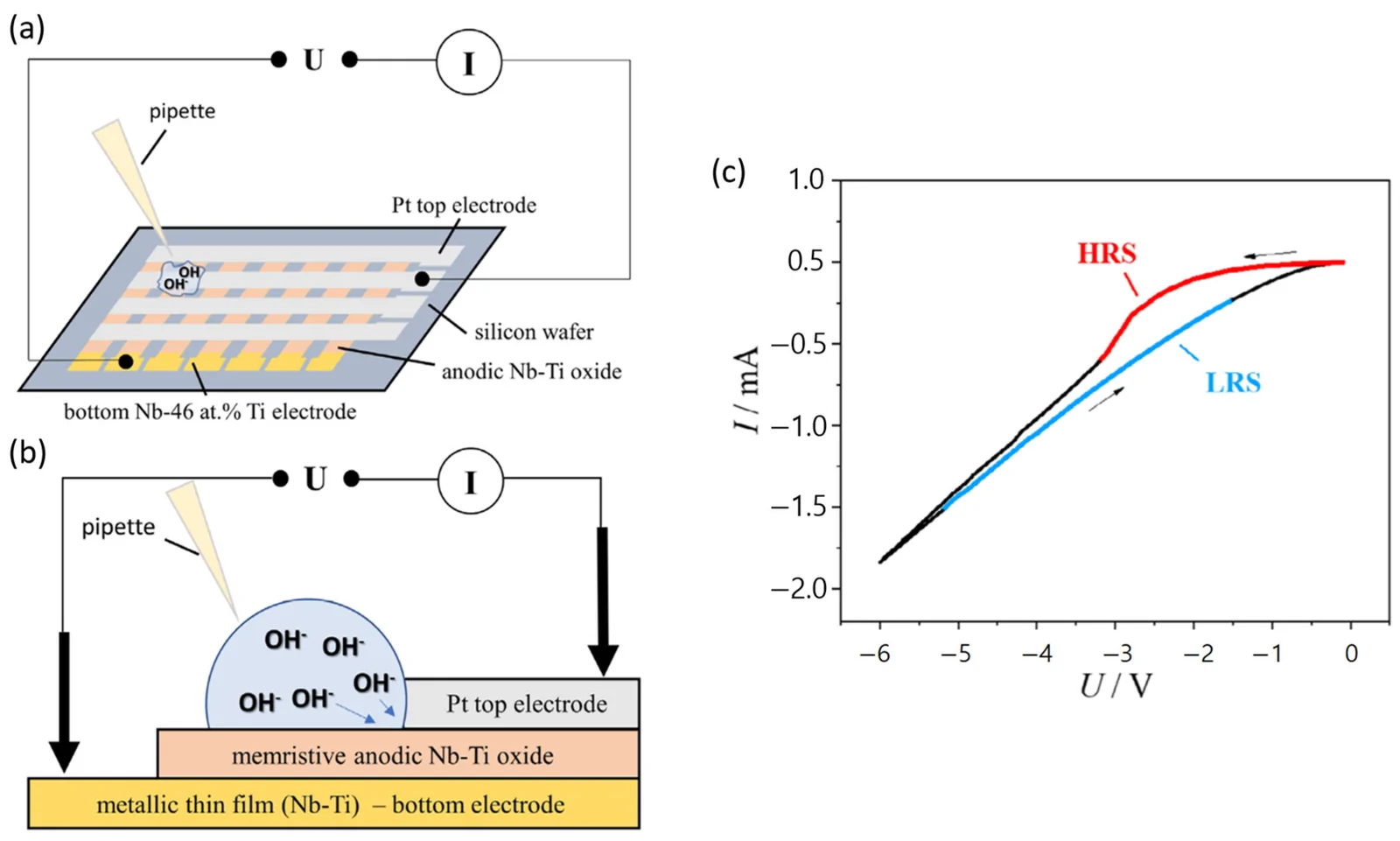
Device schematic and electrical switching behavior of the Nb-Ti oxide memristive pH sensor. The device schematic (**a**) shows the Pt top electrode, silicon wafer, anodic Nb-Ti oxide, and bottom Nb-46 at.% Ti electrode. The structural schematic (**b**) illustrates the diffusion of OH^−^ ions from the analyte into the anodic Nb-Ti oxide active layer. The corresponding I-V curve (**c**) demonstrates the pronounced hysteresis loop, highlighting the modulation of the HRS and LRS [36].

Knapic et al. [36] extended beyond conventional single-active-layer designs by employing a Nb–Ti alloy oxide as the active layer for physiological pH sensing, simulating conditions like bone implant inflammation. The device consists of a Nb–46 at.% Ti bottom electrode, an anodically formed oxide layer, and a Pt top electrode. As the pH increases, OH^−^ ions from the electrolyte diffuse into the active layer, where they either recombine with or immobilize oxygen vacancies (V_O_), thereby suppressing filament formation. Consequently, higher pH strengthens the high-resistance state, broadens the hysteresis loop, and increases the memory window, enabling clearer state discrimination. The device demonstrates in situ applicability for pH sensing, achieving a LOD of 0.4 pH and excellent linearity (r^2^ = 0.98) (Figure 4). However, several limitations should be noted from a practical application perspective. The reported on/off ratio of the memristor is relatively low (≤5), which may limit reliable state discrimination, especially in noisy or real-world environments. In addition, key reliability metrics such as device-to-device variation and cycle-to-cycle variation are not discussed, making it difficult to assess reproducibility and scalability. Furthermore, while the author reported acceptable reproducibility (3.8%) and short-term repeatability (1.4%), there is no investigation of long-term stability or endurance under repeated exposure to analytes. The lack of such degradation studies raises concerns regarding the practical deployment of the device in continuous or repeated sensing applications.

To expand the application of metal-oxide devices to metabolic monitoring, Kumar et al. [37] fabricated a cross-point memristor for urea sensing. The device sandwiches a thermally treated GdO_x_ active layer between IrO_x_ and W electrodes. Thermal annealing of the GdO_x_ layer increases the Schottky barrier height (Φ_B_) shifting from 0.39 eV to 0.59 eV, ensuring highly stable resistive switching characteristics and an improved on/off ratio. Unlike filamentary mechanisms, the sensing operation in this architecture occurs as OH^−^ ions—generated by the enzymatic breakdown of urea—penetrate the porous IrO_x_ top electrode and modulate the Schottky barrier at the internal IrO_x_/GdO_x_ interface. Changes in the surface potential induced by analyte interactions generate a measurable electrical signal. Through this mechanism, the device achieved a baseline pH sensitivity of 53.2 mV/pH and successfully demonstrated urea detection in the concentration range of 1 mM to 10 mM, monitored via distinct conductance shifts. However, despite integrating a stable memristive memory, biological sensing fundamentally relies on the enzymatic reaction of urease. This inescapable dependency fails to resolve the long-term instability and degradation issues commonly associated with enzyme-based sensors. Furthermore, the measurement setup necessitates a bulky external reference electrode to maintain a stable liquid potential, which complicates the transition toward fully integrated, scaled on-chip biosensing systems.

### 3.3. Indirect Sensing of Analytes via Memristive Devices

Direct exposure of memristors to analytes leads to irreversible degradation, effectively rendering the semiconductor-fabricated devices single-use despite their high fabrication cost. To circumvent this limitation, recent studies have proposed indirect exposure architectures.

Shulaker et al. [13] demonstrated a representative implementation of this concept using a monolithic three-dimensional (3D) nanosystem. By vertically stacking carbon-nanotube field-effect transistor (CNFET) sensors on top of a 1-Mbit memristor array at low processing temperatures (<200 °C), the sensing interface was physically decoupled from the computational core. To enable biological and chemical applicability, the CNFETs were non-covalently functionalized with materials such as DNA and metal porphyrins. The sensors converted ambient gas signals into electrical voltages, which were transmitted through inter-layer vias to modulate the memristor array, achieving a high device yield of 98.82%. This architecture protects the memristor from direct exposure to analytes while mitigating the von Neumann bottleneck by integrating sensing and memory at the device level. Meanwhile, as a prototype, it faces practical limitations. The system was implemented at a lagging 1 µm technology node, resulting in a high 3 V supply voltage and a limited operating frequency of 2 kHz.

Extending this indirect sensing paradigm to biological environments, van Doremaele et al. [19] demonstrated its feasibility in aqueous conditions. While conventional inorganic sensors are primarily optimized for gas-phase detection, biological sensing requires stable operation in liquid environments. To address this, they employed a modular co-planar integration strategy using organic materials, where the sensing module—an ion-selective organic electrochemical transistor (IS-OECT) that converts ionic signals (e.g., sweat chloride) into electronic voltages—was physically separated from the computing module, an electrochemical random-access memory (EC-RAM). This configuration enabled autonomous on-chip learning and classification operating at low input voltages from approximately ±30 mV to ±100 mV without the need for external software. Nevertheless, the reliance of the system on a single-layer perceptron algorithm limits its capability to strictly linearly separable problems, and the diagnostic validation serves as a proof of concept using modified donor sweat rather than real clinical patient data, as the authors stated.

However, despite these advances, architectures that require the sensor chip to be directly immersed in biological fluids remain fundamentally susceptible to contamination and degradation over time. Taking the indirect sensing paradigm to its physical form at the device level, Zhou et al. [14] demonstrated that direct contact between the semiconductor and the solution can be entirely eliminated. By encapsulating silicon nanowires with a 10 nm Al_2_O_3_ high-k dielectric shell, the device indirectly detects only the potential changes in target molecules. This mechanism successfully maintained stable performance for over 100 days in physiological environments of 1X phosphate-buffered saline (PBS) at 37 °C, whereas the conductance of unprotected devices degraded to less than 3% of its initial value within just 7 days.

In spite of this extended lifespan, a fundamental limitation remains: as long as the sensor chip itself must be immersed in biological fluids, it cannot completely overcome the inherent risks of prolonged exposure, rendering effective reuse impossible. Therefore, it is essential to move beyond surface chemistry and device-level encapsulation toward physically separating the sensing electrodes. To address this limitation and achieve true reusability, Kim et al. [15] proposed a fully decoupled architecture in which the gate terminal of the sensing transistor is extended externally. In this design, the analyte-contacting electrode is engineered as a replaceable, disposable module (e.g., a USB-like form factor), while the core memristive processing unit remains completely isolated, protected, and reusable. This strategy addresses the exposure issue, significantly enhancing device longevity and representing a practical pathway toward sustainable and cost-effective memristor-based sensing systems.

The representative device-level implementations discussed above are summarized in Table 1, with emphasis on sensing performance, memory/stability metrics, CMOS compatibility, and reproducibility. Taken together, these studies demonstrate that memristive-device-based biosensors and related nanowire sensing platforms can achieve high analytical sensitivity, with reported detection levels ranging from the aM–fM regime for protein or viral biomarkers to the nM level for H_2_O_2_ sensing and sub-pH-unit resolution for pH detection. However, these demonstrations remain largely at the device or proof-of-concept stage. Many direct sensing platforms achieve excellent sensitivity but still suffer from limited reusability, incomplete CMOS compatibility, insufficient long-term stability data, and a lack of commonly reported system-level metrics such as diagnostic accuracy and power consumption. In contrast, indirect and decoupled sensing architectures improve device protection and reusability by separating the core memristive element from the liquid analyte environment, although further advances in device variability control, reproducibility, and scalable integration are still required for practical commercialization.

## 4. System Level Implementations

Device-level sensors exhibit high sensitivity; however, practical deployment requires integrated signal processing blocks. Therefore, system-level implementations that integrate sensing devices with electronic circuitry are essential. The biosensor systems combine the analyte–transducer interface with a complete signal processing chain, including readout integrated circuits (ROICs), analog-to-digital converters, and digital signal processing (DSP) units. Depending on the degree of integration, these architectures can be broadly classified into off-chip and on-chip signal processing systems.

In off-chip signal processing systems, signal processing and data visualization are performed using peripheral devices, typically through wireless or wired communication modules embedded within the system. In contrast, on-chip signal processing systems execute all processing tasks within the sensor platform, enabling fully autonomous operation. Recent research trends have increasingly shifted toward internal system-level implementations to achieve higher integration density and reduced power consumption by minimizing reliance on external components.

### 4.1. Off-Chip Signal Processing System

Tzouvadaki et al. [38] implemented the traditional off -chip signal processing architecture targeting POC diagnostics for prostate cancer, specifically designed for detecting PSA at early stages. The authors developed a printed circuit board (PCB) incorporating an STM32 microcontroller unit (MCU) and a memristive biosensor array, enabling automated signal acquisition across 12 channels. This configuration made the PSA detection possible at fM concentrations while significantly reducing the measurement time from approximately 40 min to about 80 s. However, data analysis and visualization remained dependent on external devices such as a Raspberry Pi or personal computer, highlighting a key limitation of off-chip systems in terms of portability.

To address these limitations, Sun et al. [17] leveraged existing smartphone hardware as an external processing platform. Instead of integrating all components into the sensor system, the authors utilized the audio jack of the smartphone as a unified interface for both power delivery and data communication. By employing power harvesting techniques to convert audio signals into usable electrical power, they achieved a conversion efficiency of approximately 79%. Instead of relying on an on-chip analog-to-digital converter, the raw analog data was frequency-modulated using a voltage-controlled oscillator (VCO) and transmitted directly to the smartphone. Subsequently, data processing and visualization were carried out using the application processor of the smartphone and a dedicated mobile application. This system demonstrated a peak power consumption of only 6.9 mW while enabling detection of bidirectional currents below 1 nA secretory leukocyte protease inhibitor (SLPI), a biomarker for monitoring lung infections in cystic fibrosis patients, thereby validating its applicability to biological sensing in ultra-low-power and highly miniaturized platforms without dedicated batteries. Despite these advantages, this off-chip architecture remains fundamentally dependent on an external centralized device (i.e., the smartphone) for core computational tasks. Continuous data transfer between the sensor node and the mobile processor introduces communication latency and additional energy overhead. This reliance on data movement highlights the inherent von Neumann bottleneck, indicating that offloading computation to a portable external device does not resolve the fundamental energy inefficiencies associated with separated sensing and processing.

### 4.2. On-Chip Signal Processing System

**Figure 5:**
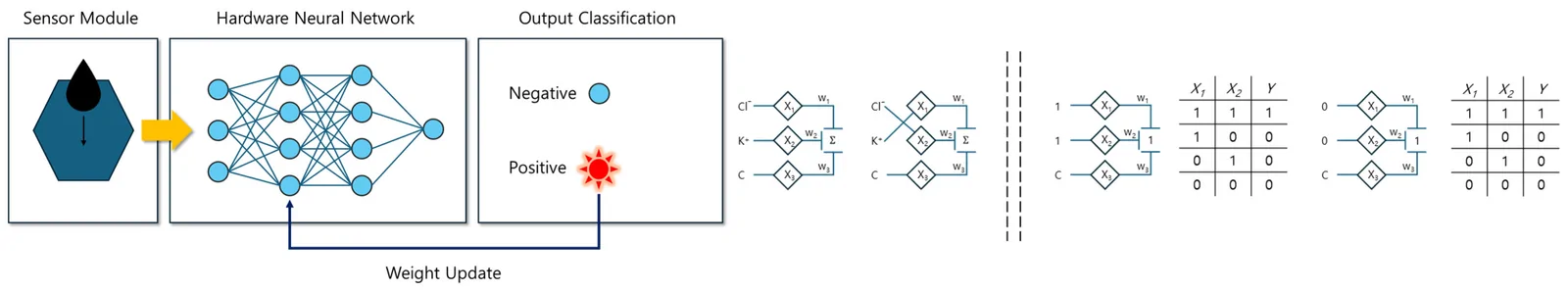
Autonomous internal signal processing system utilizing organic electronic materials. The system schematic (**left**) illustrates the modular neuromorphic architecture, integrating an ion-selective sensor module directly with a hardware neural network and output classification stage. The corresponding experimental data (**right**) demonstrates the successful execution of hardware-based on-chip learning, highlighting dynamic weight updates across multiple training iterations for accurate classification [19].

In contrast, on-chip signal processing systems perform all signal processing tasks within the sensor platform, eliminating the need for external computational units. Van Doremaele et al. [19] demonstrated such an architecture by integrating sensing, computation, and classification functionalities on a single substrate using organic electronic materials. Their system incorporated IS-OECTs, EC-RAM, and a classification output stage, enabling real-time decision-making directly at the sensor level. The conductivity of EC-RAM devices is modulated by error signal feedback during the learning phase, enabling on-chip learning and adaptation. The system achieved disease classification, such as cystic fibrosis diagnosis, with approximately 19 training iterations in specific configurations utilizing commercial sensors integrated with an offset circuit to scale the input signals. This process demonstrated reliable performance using modified donor sweat samples simulating pathological chloride measurements (>60 mmol/L). The linear and symmetric conductance modulation of the EC-RAM devices minimized quantization errors during training, enabling accurate classification when integrated with an offset circuit to scale sensor outputs. Owing to its low-voltage operation and compact integration, this approach is well suited for POC diagnostics and wearable applications (Figure 5). However, despite successfully demonstrating localized learning and classification, the reliance on organic electronic materials presents challenges for large-scale commercialization. Organic devices are inherently incompatible with CMOS fabrication processes, limiting scalability and manufacturing yield. These limitations suggest a transition toward CMOS-compatible, inorganic in-sensor computing platforms for practical deployment.

Although the aforementioned off-chip and on-chip platforms have successfully advanced biosensing capabilities, they share a fundamental architectural limitation: the physical separation between the sensing interface and the computational unit. Whether relying on extended metal lines to extract signals from microfluidic chips as presented by Vallero et al. [16] or coupling distinct sensor and memory modules as proposed by Van Doremaele et al. [19], these systems necessitate continuous data transfer. To fundamentally transcend the energy and latency constraints of this von Neumann bottleneck, architecture must move beyond physically separated components and evolve toward a neuromorphic in-sensor computing paradigm.

In this context, Khalid et al. [18] highlighted the convergence of hardware and Artificial Intelligence (AI) as a critical pathway for real-time, on-site decision-making. By incorporating Edge AI, this approach drastically reduces data transfer overhead and power consumption. Furthermore, they examined the concept of physical reservoir computing, which exploits the intrinsic nonlinear dynamics of the sensor devices themselves to perform computation, effectively blurring the physical boundary between signal transduction and information processing.

To transform these neuromorphic ideals into a scalable reality, Tintelott et al. [39] emphasized the necessity of CMOS-compatible inorganic materials to leverage existing semiconductor infrastructure. By combining the high-volume manufacturability of CMOS with top-down standardized processes like nanoimprint lithography (NIL), the strict device-to-device uniformity required for large-scale neuromorphic arrays can be ensured. Ultimately, the integration of data-stationary in-sensor AI with robust CMOS-compatible hardware represents the most definitive evolution toward sustainable and intelligent biosensing systems.

These representative system-level implementations are summarized in Table 2. Overall, these studies reviewed in this section illustrate the system-level transition from externally assisted readout platforms toward more integrated on-chip signal-processing architectures. Unlike device-level demonstrations, these works are better compared in terms of implementation status, readout architecture, external processing dependence, power consumption, measurement time, and system-level functionality, together with available analytical metrics.

## 5. Broadening the Paradigm: In-Sensor Computing

**Figure 6:**
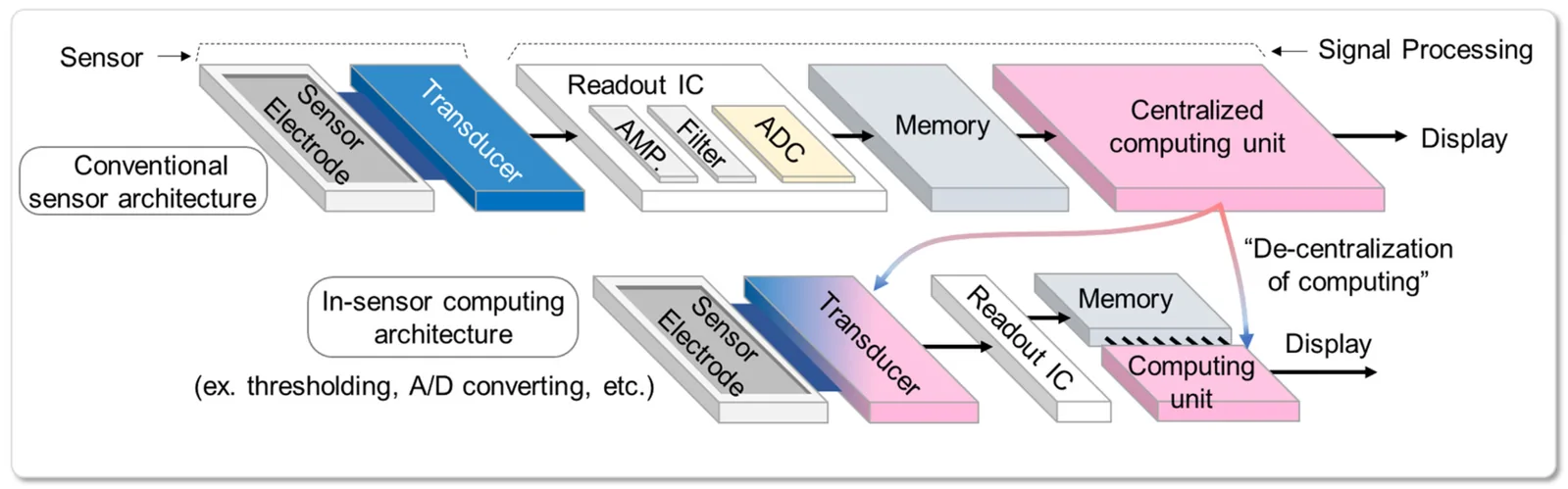
Conceptual comparison between the conventional von Neumann sensor architecture and the proposed in-sensor computing paradigm. The schematic illustrates the decentralization of computational workload shifting from a power-intensive centralized computing unit to an ‘in-sensor computing’ architecture, where fundamental data processing tasks (e.g., thresholding, data conversion) are embedded directly at the transducer level to circumvent data migration bottlenecks.

Conventional biosensor systems are predominantly based on the von Neumann architecture, in which sensing and signal processing units are physically separated. This separation necessitates continuous data transfer between the transducer and processing units, leading to increased latency and energy consumption. To mitigate this, recent studies have demonstrated the feasibility of offloading pre-processing tasks, such as convolution operations, directly to the sensor edge through reconfigurable hardware frameworks [6,40]. To overcome these limitations, recent research has increasingly focused on in-sensor computing, a paradigm in which sensing and computation are co-localized within the same physical node [7]. By integrating these functions, in-sensor computing minimizes data movement and enables more efficient processing in terms of energy, speed, and system footprint. At the device level, this approach extends beyond conventional transducer operation by embedding computational functionality directly into the sensing unit. As a result, sensing and inference can be performed simultaneously without relying on external processing blocks [41]. Figure 6 illustrates the conceptual block diagram of the in-sensor computing architecture compared to the conventional von Neumann system.

### 5.1. Device-Level In-Sensor Computing: Physical Integration

Device-level integration of in-sensor computing strictly aims to combine sensing and primary computation within a single physical unit. Recent advancements have demonstrated this hardware integration across various sensory domains by enabling local decision-making and feature extraction at the single-device level.

**Figure 7:**
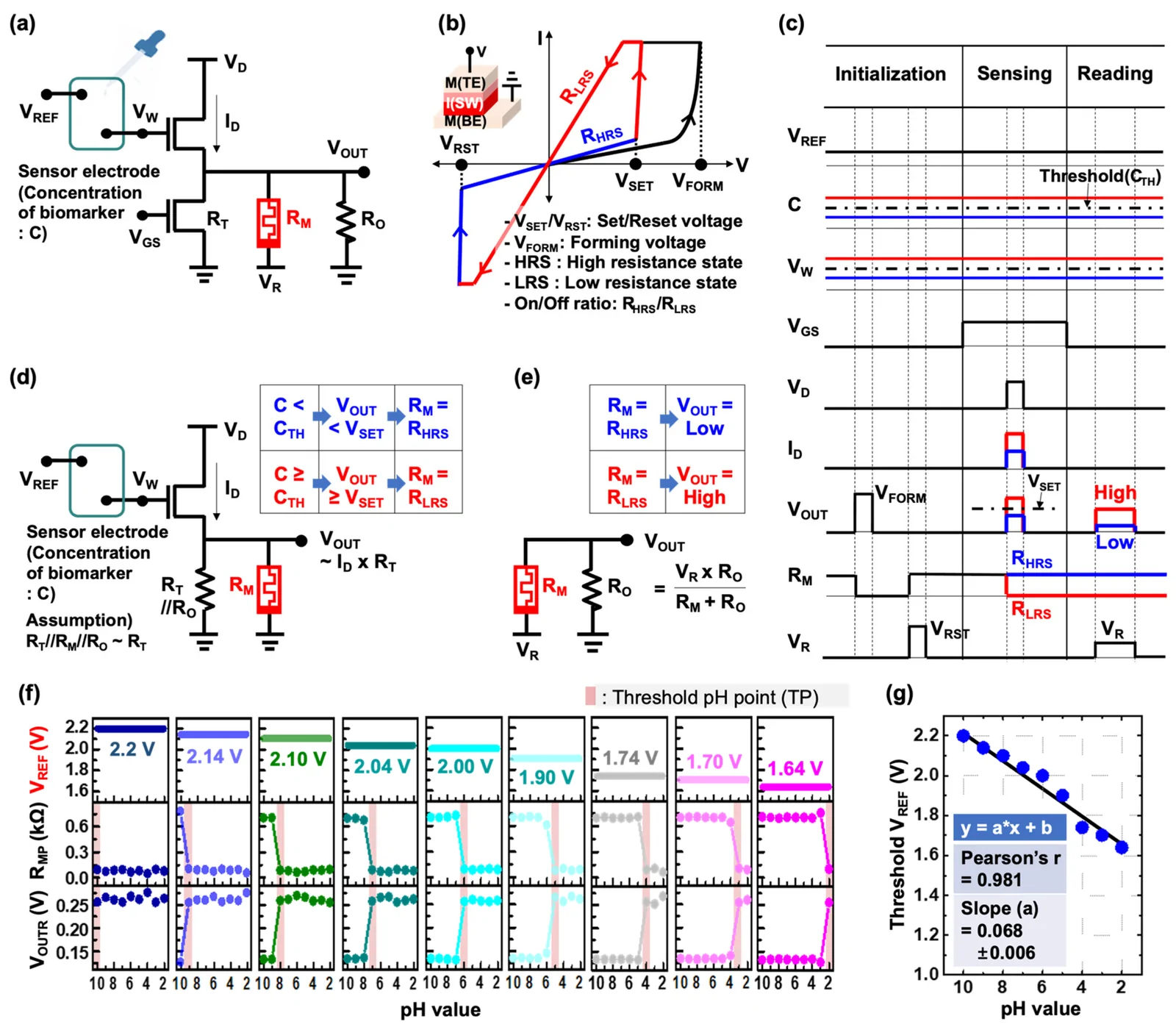
Operating mechanism and experimental verification of the CMOS-compatible in-sensor computing memristive biosensor [15]. (**a**) Circuit schematic of the transducer integrating a sensor electrode, two FETs, a memristor (R_M_), and an output resistor (R_O_). (**b**) Typical I-V characteristics of the memristor, illustrating the switching between the HRS and LRS switching at the threshold voltages (V_SET_ and V_RST_). (**c**) Timing diagram showing the sequential operation across Initialization, Sensing, and Reading phases. (**d**) Operation principle of Sensing phase: the biomarker concentration (C) dictates the induced voltage (V_OUT_), which directly determines the resistance state of the memristor depending on whether it exceeds V_SET_. (**e**) Operation principle of Reading phase: the memristor resistance state (R_HRS_ or R_LRS_) is converted into a digital logic output (Low or High) for in-sensor computing system. (**f**) Experimental demonstration of highly programmable threshold sensing: modulating the reference voltage (V_REF_) actively shifts the target threshold pH point (TP) across the entire pH spectrum. (**g**) The extracted linear correlation between the threshold V_REF_ and the target pH value, exhibiting exceptional reliability (Pearson’s r = 0.981) [15].

In the biochemical domain, Kim et al. [15] demonstrated a representative device-level implementation of an in-sensor computing biosensor by integrating a sensor electrode, two FETs, a TaO_x_/Ta_2_O_5_-based memristor, and an output resistor into a transducer. In this configuration, the upper FET converts the electrochemical potential at the sensor electrode into a drain current, which is subsequently translated into an output voltage through a variable resistance element. When the output voltage exceeds the switching threshold of the memristor, the device undergoes a transition from an HRS to an LRS, enabling threshold-based sensing and decision making in the transducer (Figure 7a–e). The biosensor enables tunable threshold-sensing functionality, allowing the detection of different analyte conditions within a single platform by adjusting the integrated reference voltage. Notably, threshold points corresponding to pH values from 2 to 10 were demonstrated with a linear response (Pearson correlation coefficient of 0.981), indicating reliable and tunable sensing performance (Figure 7f,g). Furthermore, to mitigate degradation caused by direct exposure to electrolyte environments, the sensing electrode was implemented as a replaceable external module, allowing the core memristive unit to be reused.

**Figure 8:**
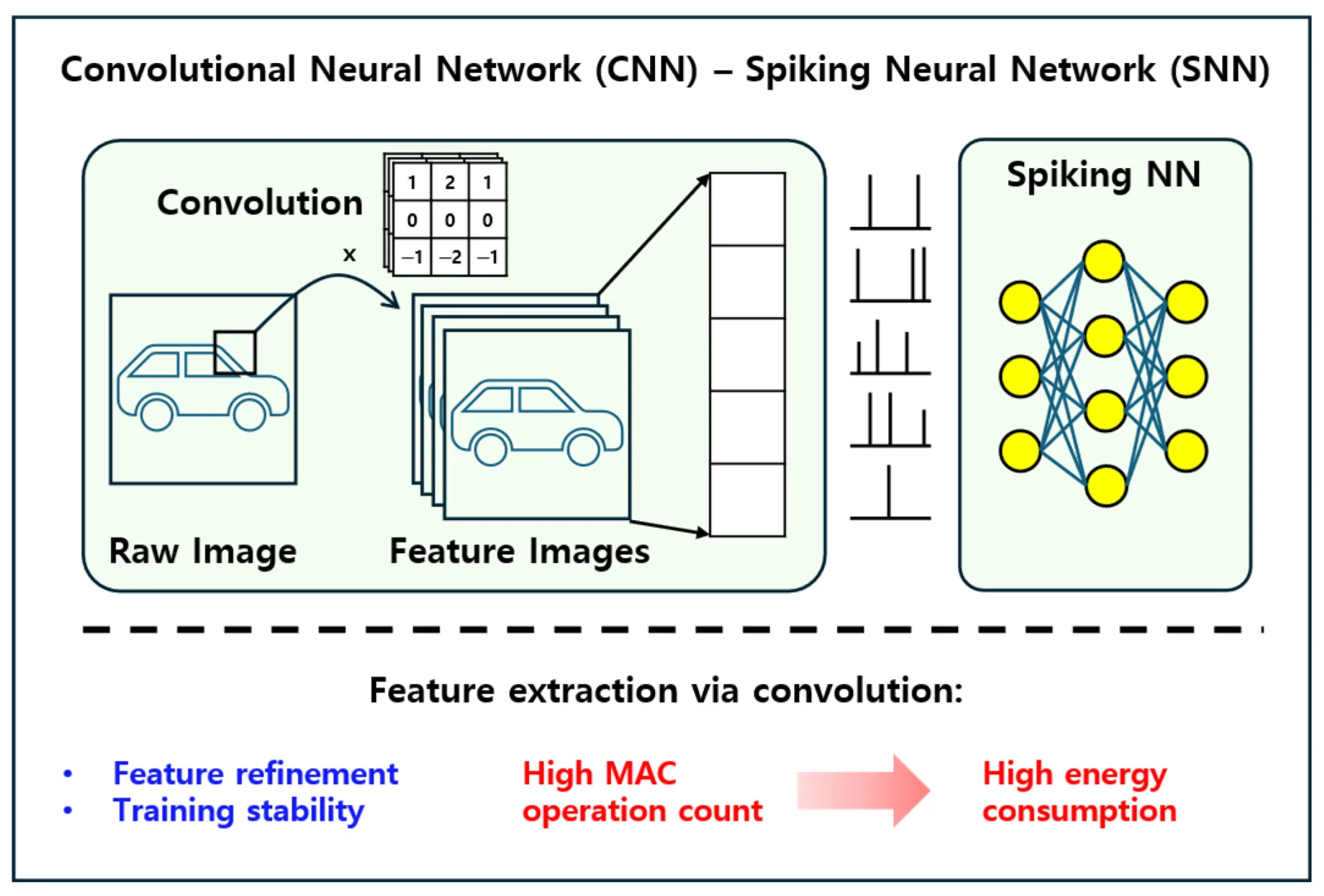
Schematic overview of the Convolutional Neural Network (CNN)–Spiking Neural Network (SNN) processing pipeline. The schematic illustrates feature extraction via convolution and high-energy MAC operations. Reprinted with permission from [20].

Extending this concept to the visual domain, Kim et al. [20] presented a monolithic 3D architecture combining an amorphous silicon (a-Si) PIN photodiode array with a polysilicon thin-film transistor (TFT)-based spiking circuit. In this device, analog multiply-and-accumulate (MAC) operations are physically performed within localized 3 × 3 pixel clusters. This structure enables spatial filtering operations, such as blurring and smoothing, directly at the sensor interface without the need for intermediate digital conversion. Furthermore, the device directly converts continuous wave illumination (0.1 to 12 mW/cm^2^) into frequency modulated spike trains (200 Hz to 1.2 kHz). By performing feature extraction in the analog domain, this hardware level integration significantly reduces data transfer overhead while preserving essential image features under varying light conditions (Figure 8).

**Figure 9:**
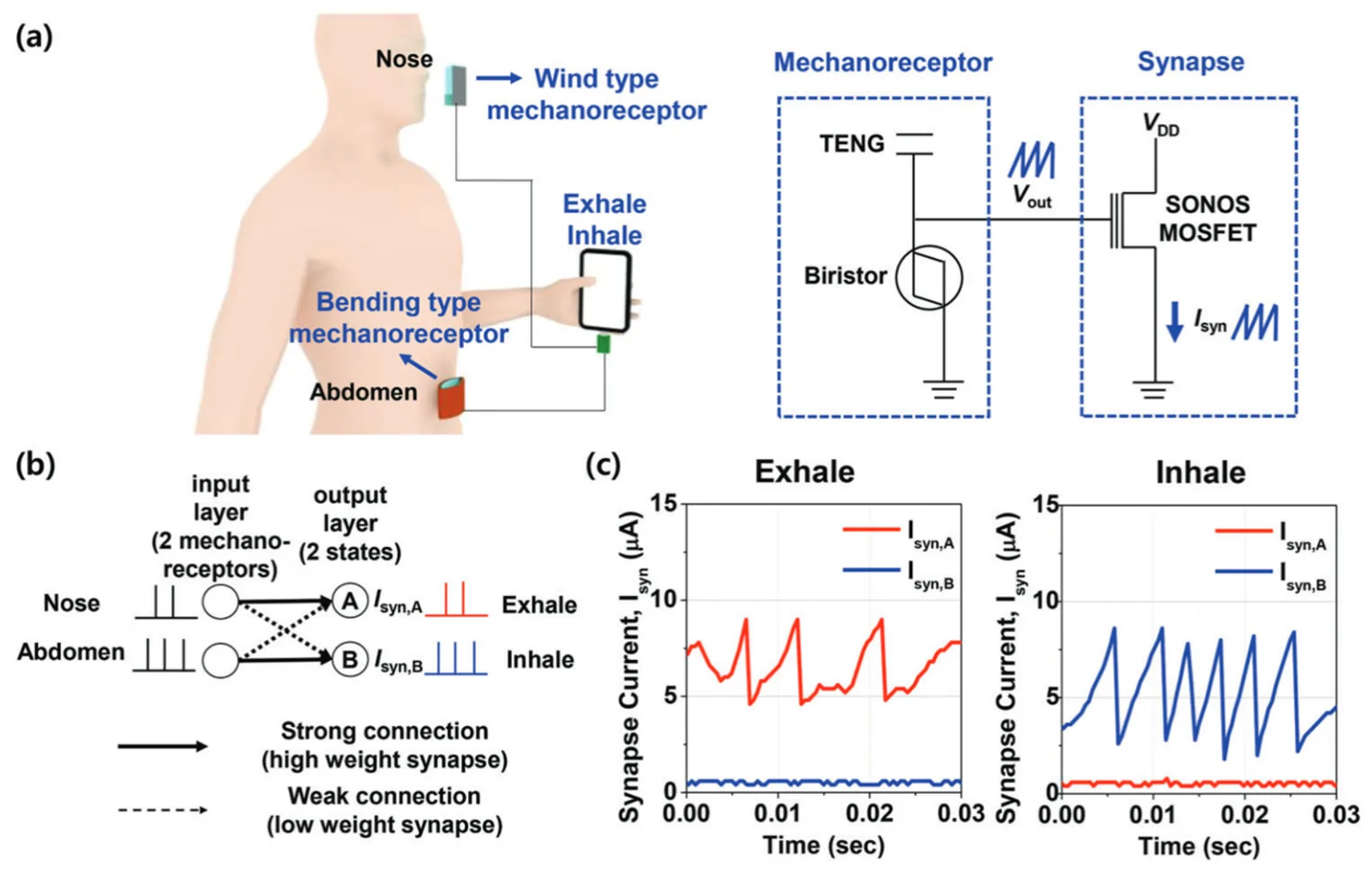
Self-powered artificial mechanoreceptor system and its application in human breath monitoring. (**a**) Schematic and circuit diagram of the self-powered mechanoreceptor system integrating wind-type and bending-type TENG sensors with a biristor-based neuron unit. (**b**) Synaptic connection map between the input mechanoreceptors and the output layer, using two types of arrows to represent the distribution of synaptic weights for respiratory state classification. (**c**) Detected synapse current data (Isyn,A and Isyn,B) for exhale and inhale phases, showcasing the real-time pattern recognition capability of the integrated neuromorphic system [21].

Similarly, exploring the tactile domain, Han et al. [21] demonstrated a self-powered artificial mechanoreceptor by integrating a triboelectric nanogenerator (TENG) with a biristor-based neuron device. (Figure 9) This system represents a highly energy efficient architecture where mechanical stimuli are directly converted into spike-based signals without relying on external power sources. By utilizing this self-powered sensing and computation scheme, the physical device successfully achieved pressure detection down to near 3 kPa. Furthermore, they successfully demonstrated a fully hardware-based breath-monitoring system capable of classifying exhalation and inhalation using wind- and bending-type mechanoreceptors, highlighting its potential for continuous tactile monitoring.

### 5.2. Array-Level In-Sensor Computing: From Temporal Dynamics to Spatiotemporal Vision

To process complex, real-world data streams, in-sensor computing must evolve beyond individual units. According to Wan et al. [7], array-level computing physically couples sensor nodes to form receptive fields and perform parallel MAC operations, effectively implementing artificial neural networks (ANNs) directly at the hardware level. This evolution unfolds in three progressive tiers: establishing foundational temporal dynamics, processing one-dimensional (1D) physiological signals, and executing complex two- and three-dimensional (2D/3D) spatiotemporal visual tasks.

#### 5.2.1. Basic Temporal Dynamics and Reservoir Computing

To achieve high-dimensional computation, arrays leverage the inherent time-dependent physics of memristive materials. Beyond static weight storage, integrated memristor networks have also demonstrated physiological signal processing and reconfigurable neuromorphic sensing with hardware vector–matrix multiplication readout, highlighting their capability for higher-level signal processing [42,43]. As Park et al. [44] noted, physical Reservoir Computing (RC) exploits fading memory and non-linear mapping to process time-series data with extreme energy efficiency. Experimentally, Park et al. [45] demonstrated a highly uniform Pt/TiO_x_/Ti crossbar array that provides a hardware foundation for temporal sequence processing. The volatile decay dynamics—governed by the activation energy of oxygen anions—endows the array with an inherent fading memory that functions as a structural temporal kernel, successfully learning the structural grammar of antimicrobial peptides. Li et al. [46] and Lin et al. [47] further validated that stochastic phase-change volatility autonomously substitutes explicit recurrent loops for time-delayed data. Shim et al. [48] advanced this by proposing a two-memristor one-capacitor (2M1C) temporal kernel where dual memristors extract complementary spatiotemporal features, enhancing signal separability. Bridging sensing and memory, Syed et al. [40] integrated phase-change memory (PCM) directly into sensor pixel arrays, utilizing reconfigurable load lines to execute parallel convolutions entirely during the data acquisition phase.

#### 5.2.2. Macroscopic Trends in 1D Bio-Signals

**Figure 10:**
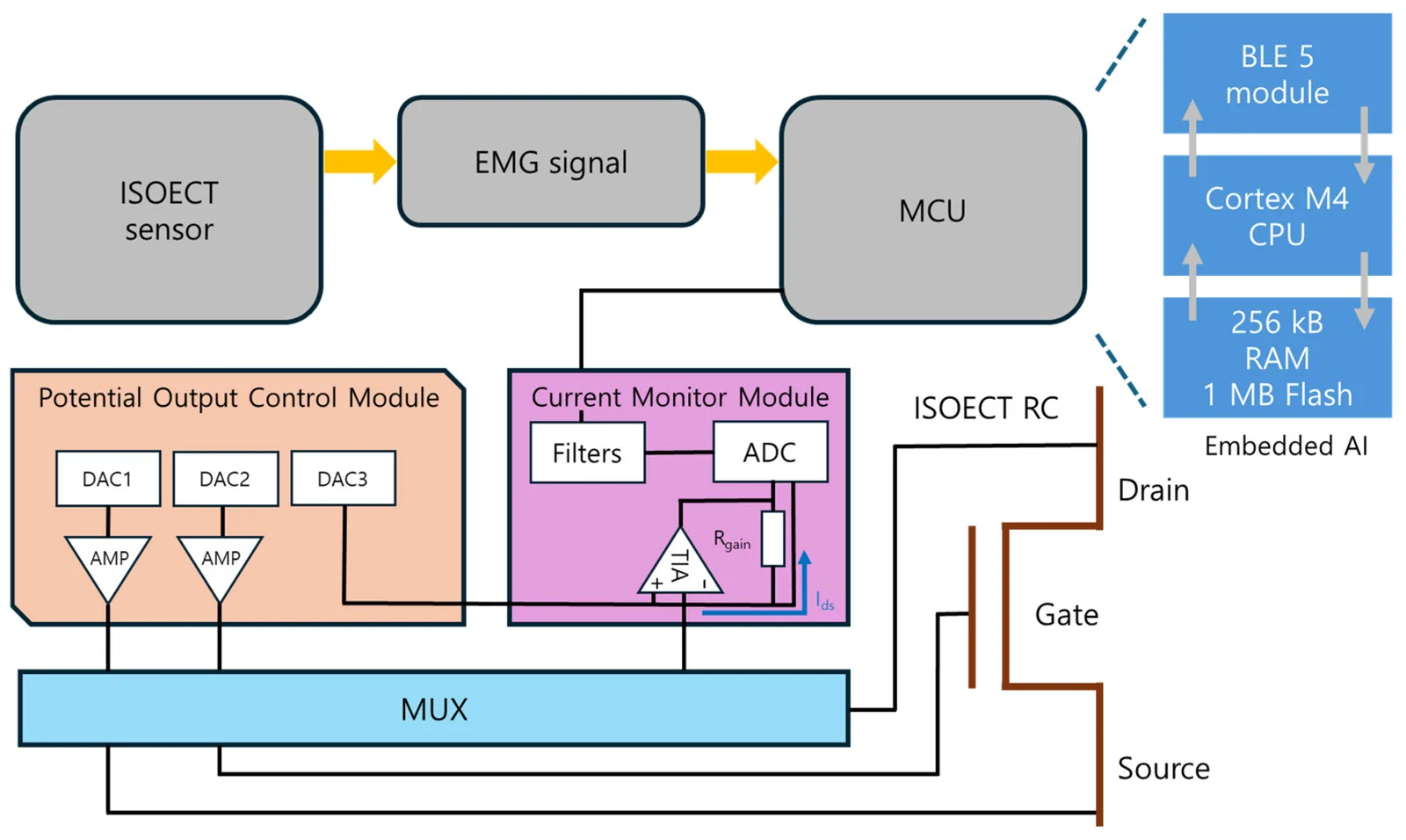
The diagram illustrates the overall data flow of the wearable gesture recognition system, consisting of the stretchable sensor array, the external MCU for signal digitization and pulse encoding, and the organic electrochemical transistor (OECT) based reservoir for computation [22].

Building on these physical temporal kernels, arrays have been aggressively deployed for macroscopic 1D physiological monitoring. Demonstrating robustness in clinical diagnostics, Zhang et al. [49] implemented a heterojunction Ag/ZnWO_4−x_/WO_3−x_/ITO optoelectronic memristor architecture that mapped sequential electrocardiogram (ECG) waveforms into discrete time-step intervals, achieving an onsite arrhythmia detection accuracy of 96.84%. Similarly, Zhang et al. [50] mapped multimodal raw electroencephalogram (EEG) and snoring acoustic streams using textile-integrated fiber memristor arrays to autonomously monitor sleep stages. Furthermore, De Luca et al. [51] proposed a neuromorphic architecture utilizing the DYNAP-SE chip, which enables multi-time scale heart rate decoding and local motion artifact rejection without software-heavy algorithms. However, array implementations still face bridging challenges; for instance, Liu et al. [22] developed a stretchable organic electrochemical transistor (OECT) array for electromyography (EMG) gesture recognition (achieving 90% accuracy at <0.56 nJ/operation) as shown in Figure 10, but the architecture still inherently relied on an external MCU for digitizing analog inputs into pulse streams, highlighting the ongoing necessity for fully autonomous, MCU-independent architectures.

#### 5.2.3. Spatiotemporal Video and Camera Applications

The culmination of array-level computing targets the most data-intensive bottleneck: spatiotemporal 2D/3D visual processing. Addressing this, Wang et al. [52] emphasized retina-inspired optoelectronic memristors (OEMs) that physically mimic the human visual pathway, filtering 80% of redundant data directly at the sensing array. Adding active feedback to such vision arrays, Hou et al. [53] proposed a multifunctional micro-light emitting diode (LED) device integrated with neuromorphic processing capable of executing learning-forgetting-relearning cycles for energy-efficient intelligent displays. The definitive milestone in this tier is presented by Jeong et al. [54] who developed a selector-less memristor array capable of executing self-supervised video stream processing entirely on-chip. By deploying on-device gradient descent training, the hardware dynamically calibrates spatial device variability and parasitic wire resistance. This enables real-time background and foreground video separation without external pre-training, achieving a peak signal-to-noise ratio (PSNR) of 30.49 dB, thereby proving the viability of autonomous spatiotemporal processing at the near-sensor edge. However, a critical distinction must be drawn between real-time inference and continuous real-time learning. While current memristive arrays, which typically require weight update pulse widths of approximately 1 µs, are highly capable of executing real-time inference based on pre-established parameters, continuous on-device training under high-frame-rate visual inputs remains a formidable physical challenge. To fully unlock the potential of autonomous, lifelong learning at the sensor edge, systematically scaling the hardware weight update time down to the sub-microsecond or nanosecond regime is an imperative next step, marking a critical frontier for future device engineering.

### 5.3. System-Level In-Sensor Computing: Architecture Integration

While array-level networks efficiently handle data processing bottlenecks, achieving true practical deployment requires system-level integration. This involves eliminating external MCU dependencies, achieving extreme high-density packaging, and introducing novel structural form factors to support fully autonomous bio-platforms.

Innovations in form factor and spatial configurations are driving this system-level evolution. Costa et al. [55] addressed high-density integration limits by developing a vertically designed mesh micro-chip utilizing a single-response multiplexing (SERM) strategy. By employing dual quasi-reference electrodes (Au and Ag/AgCl), the system eliminated spatial crosstalk using a single redox probe, doubling the assay throughput for precise COVID-19 antibody screening. Advancing physical adaptability, Merces et al. [56] introduced 4D microelectronics based on origami tessellation. These morphogentronic systems self-fold in response to environmental stimuli (e.g., pH or electrical actuation), creating highly adaptive, bio-inspired smart stents and energy storage devices. Furthermore, to overcome the limitations of direct skin contact, Shin et al. [57] introduced a non-contact wearable platform measuring epidermal molecular flux (such as VOCs and CO_2_) through programmable magnetic valves, enabling continuous wireless tracking of wound healing and moisture homeostasis without skin irritation.

**Figure 11:**
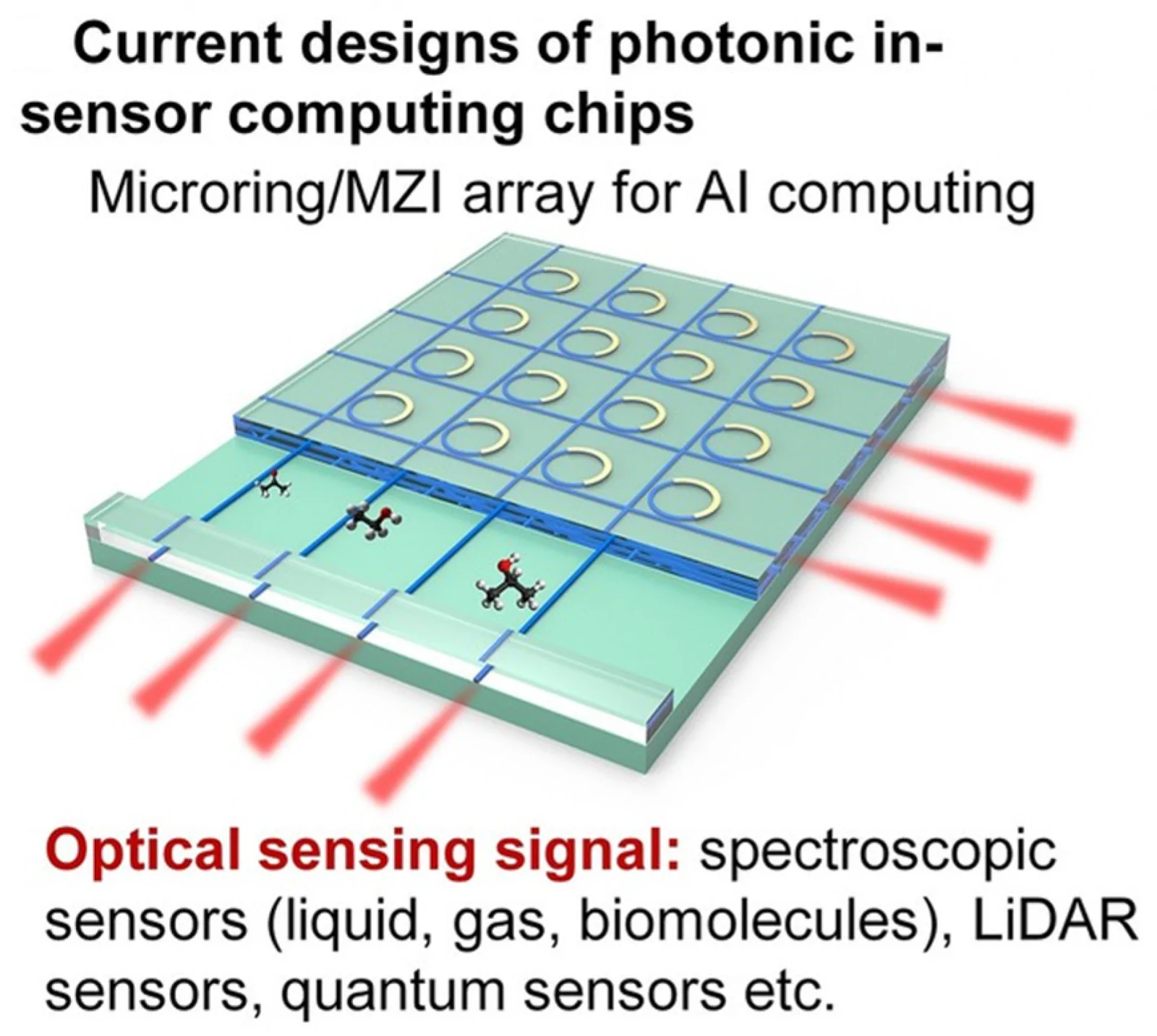
Illustration of hybrid photonic-electronic in-sensor computing chips utilizing microring and MZI arrays [23].

Beyond structural ingenuity, the ultimate pursuit of system-level speed and energy efficiency is transitioning toward heterogeneous photonic-electronic hybridization. Ren et al. [23] demonstrated a CMOS-compatible near-sensor computing platform utilizing bilayer AlN/Si waveguides. Unlike conventional electronic systems that suffer from analog-to-digital converter (ADC) and digital-to-analog converter (DAC) conversion delays and MCU overhead, this platform physically offloads MAC operations to Si Mach-Zehnder interferometers (MZIs) in the optical domain, as shown in Figure 11. By mitigating the electronic data movement bottleneck, the system achieved complex tasks, such as gesture and gait recognition, with ultra-low latency (<10 ns) and minimal energy consumption (<0.34 pJ). This holistic integration of macroscopic form-factor adaptability, wireless autonomy, and optical-domain processing represents the ultimate frontier of fully decentralized, system-level neuromorphic biosensing architectures.

To clarify the device-to-array-to-system progression discussed in Section 5, Table 3 summarizes selected in-sensor computing platforms and related system-level sensing architectures across device-, array-, and system-level implementations. Rather than applying a single biosensor-specific metric to all platforms, the table compares the input domain, device platform, integrated operation, and quantitative performance metrics according to the functional role of each system. This organization distinguishes the electrochemical threshold-sensing biosensor from optical, tactile, electrophysiological, wearable, and multiplexed sensing platforms, while highlighting transferable strategies for integrating sensing with local memory, computation, or readout functions.

Overall, Table 3 illustrates a stepwise architectural expansion of in-sensor computing, from single-device thresholding and feature extraction, through array-level temporal and spatiotemporal processing, to system-level autonomous or high-throughput sensing architectures. This progression shows that practical in-sensor computing for biosensing cannot be achieved by device innovation alone, but requires coordinated advances in array scalability, peripheral integration, power efficiency, and system-level form factor. At the same time, the heterogeneous metrics indicate that electrochemical in-sensor biosensors remain at an early convergence stage, requiring improved clinical validation, variability-tolerant multi-threshold sensing, and long-term stability. These remaining gaps motivate the enabling technologies discussed in the next section.

## 6. Future Perspectives: Enabling Technologies

Building upon the systematic structural evolution of memristive biosensors established in the preceding sections, the following section delineates emerging technological trajectories. These future perspectives explore novel frontiers—such as low-dimensional materials, ion-gating vertical transistors, computationally guided biosensor discovery, bio-hybrid energy systems, and transient electronics—that transcend the limitations of current inorganic integration. Recent studies have also reported ion-gating vertical transistors as low-power neuromorphic devices and computationally guided biosensor discovery pipelines for rational recognition-element design [58,59].

Although traditional metal-oxide memristors provide robust CMOS compatibility, the pursuit of ultra-low power consumption and atomic-scale miniaturization is driving the exploration of 2D materials. Chen et al. [60] highlighted that 2D materials uniquely support diverse physical mechanisms for in-sensor computing, including charge density waves (CDW) for picosecond-level switching and spintronic devices utilizing topological skyrmions for high-density information processing. Furthermore, hybrid 2D configurations offer unprecedented control over synaptic plasticity. For instance, He et al. [61] demonstrated a bilayer graphene-based electrochemical random-access memory (EC-RAM) that optimizes Li^+^ ion intercalation through specific geometric pore designs, significantly expanding the dynamic range of artificial synapses. By combining such 2D heterostructures with foundational memristive architectures, future platforms can achieve superior cut-in threshold properties and noise suppression, as envisioned by Prajapati et al. [26].

As neuromorphic sensors transition toward continuous in vivo monitoring, absolute energy autonomy becomes a critical requirement. Moving beyond the conventional integration of external batteries or simple piezoelectric generators, research is advancing toward complex bio-hybrid energy harvesting. Egunov et al. [62] presented a groundbreaking paradigm by developing a 3D coaxial Swiss-roll microbial fuel cell (MFC) utilizing strain-induced self-assembly. By physically isolating the microbial metabolism from the power-generating interface, this architecture effectively mitigates biofouling—a persistent challenge in biological environments—while achieving a high volumetric power density of ~3.1 mW/cm^3^. Integrating such self-assembled, bio-metabolic power sources directly with ultra-low-power memristive arrays could realize fully autonomous, self-sustaining implantable diagnostic systems.

The ultimate trajectory for implantable in-sensor computing nodes involves transient electronics that seamlessly integrate with biological tissues and gracefully degrade after completing their diagnostic lifecycle. Lan et al. [6] emphasized that incorporating organic and biodegradable materials into memristive frameworks resolves the mechanical mismatch between rigid inorganic chips and soft biological tissues. These highly conformable, tissue-like intelligent systems promise to execute localized closed-loop therapeutic interventions—such as epileptic seizure prediction or real-time neurotransmitter monitoring—and subsequently dissolve harmlessly within the body. This convergence of neuromorphic computing capability with absolute biocompatibility represents the most profound frontier for next-generation medical electronics.

## 7. Discussion

Building on the device-, array-, and system-level implementations reviewed above, the remaining question is how these demonstrations can be translated into reliable biosensing systems. Although in-sensor computing can reduce data movement by embedding thresholding, feature extraction, and temporal processing close to the sensing interface, current platforms remain constrained by material/process compatibility, device variability, threshold margin, and scalable array-to-CMOS integration. The following discussion therefore focuses on the practical bottlenecks that must be resolved for memristive-device-based electrochemical biosensors to progress from laboratory prototypes to point-of-care and wearable systems: device-level robustness and reusability, system-level calibration, reliable multi-threshold sensing operation, and array-level manufacturability.

### 7.1. Device-Level Considerations

To ensure practical viability, memristive-device-based biosensors must be compatible with standard CMOS fabrication processes to enable scalable and cost-effective manufacturing. Back-end-of-line (BEOL)-compatible oxide memristor stacks, such as TaO_x_/Ta_2_O_5_, provide a promising pathway for combining process compatibility, switching stability, and circuit-level threshold-sensing operation. However, device variability remains a central challenge for reliable threshold-based biosensing. Representative memristive devices have exhibited cycle-to-cycle V_SET_ coefficients of variation ranging from approximately 9% to 32% under repeated switching operation [63,64]. Although Kim et al. [15] limited the influence of resistance variability on threshold-sensing reliability through a parallel memristor-cell configuration, improving the intrinsic uniformity of V_SET_ through device engineering such as filament-confinement strategies would further enhance sensing reliability and reduce the need for variation-compensation circuitry, thereby facilitating higher system integration density [65].

In addition, device reusability and circuit-level robustness are important considerations. While direct exposure of memristive devices to analytes can lead to irreversible degradation, indirect exposure architectures—where only a disposable sensing interface is in contact with the analyte—can preserve the integrity of the core device and improve long-term usability. Furthermore, to encompass inherent V_SET_ variations, transducer circuit optimization is strictly required to prevent signal attenuation. For instance, in conventional series-connected transistor pairs, an initial sensing signal of approximately 57 mV/pH can significantly degrade to ~26 mV, narrowing the margin for reliable threshold switching. To overcome this, recent implementations demonstrate that optimizing the gate-bias of the lower transistor and increasing the gate width ratio between the upper and lower transistors to more than 10 can critically enhance the transconductance (gM). This device-level design mitigates signal loss, securing an output voltage margin exceeding 50 mV, which ensures that the sensing signal remains distinguishable even in the presence of stochastic memristor switching. Furthermore, improving energy efficiency is a primary motivation for the transition toward in-sensor computing. At the device level, developing memristive devices operating in the low-current regime (pA–nA) is critical for minimizing overall power consumption. 

### 7.2. System-Level Considerations

Variability in device characteristics remains a key challenge at the system level. To mitigate this issue, the integration of comprehensive calibration strategies and system-level algorithms is required to address both inter-device and intra-device variations. Since V_SET_ variation generally follows a Gaussian distribution, inter-device reliability can be achieved by applying sensor trimming techniques. This involves precisely adjusting the reference voltage (V_REF_) to compensate for average V_SET_ offsets among different devices, effectively confining the operational distribution within a ±1 standard deviation range. Simultaneously, inherent intra-device variations must be managed through algorithmic approaches, such as repeated-measurement and averaging protocols, where multiple iterations are executed and statistical outliers are dynamically discarded.

These dual-track approaches reduce the dependence on strict hardware uniformity, and adaptive techniques, such as on-chip learning and feedback-based weight updates, can further help maintain stable system performance. By combining these systemic algorithms with the widened output voltage margins, ultra-low-power architectures can potentially achieve error-free threshold detection within a strict interval of one pH unit, enhancing practical reliability. Moreover, achieving ultra-low-power operation also requires architectural optimization. Energy-efficient circuit designs are essential to realizing further reductions in energy usage, potentially exceeding an order of magnitude compared to conventional architectures, which remains an important research direction.

### 7.3. Advancing the Functionality of In-Sensor Computing

Most current electrochemical biosensors operate based on binary (1-bit) threshold sensing, which is sufficient for basic detection but limits quantitative analysis. To enable more advanced diagnostic capabilities, the development of reliable multi-threshold sensing operation is necessary. In the CMOS-compatible memristor-based electrochemical biosensor transducer, programmable threshold tuning has already been demonstrated by shifting the threshold pH point from pH 10 to pH 2 in one-pH-unit increments through V_REF_ control [15]. Therefore, the next functional challenge is to transform this capability into variability-tolerant and error-free multi-threshold sensing operation under practical V_SET_ distributions.

A central requirement is statistical quality control of memristor variability. Because V_SET_ variation in memristive devices generally follows a Gaussian distribution, devices located in the distribution tail can degrade diagnostic accuracy even when the average threshold condition is properly calibrated. Future systems should therefore integrate low-variation memristors, widened ΔV_OUT_ margins, and system-level algorithms that preferentially utilize devices confined within a ±1σ V_SET_ distribution. In this framework, V_REF_-based sensor trimming can compensate for average V_SET_ offsets among devices, while repeated measurement, averaging, and outlier rejection can suppress intra-device fluctuations. By combining these approaches, demonstrated pH-unit-level threshold tuning can be advanced toward error-free threshold-based classification, and subsequently toward finer sub-unit diagnostic resolution if sensor-electrode-induced slope deviation, noise, and V_REF_ control precision are further improved.

Ultimately, mastering reliable multi-threshold sensing operation under strict ultra-low-power constraints (pA–nA) will be essential for next-generation form factors. As highlighted by emerging research [20,21], resolving these challenges will facilitate the transition from external wearable platforms to fully autonomous, battery-independent ingestible and in vivo monitoring systems. In such environments, continuous and quantitative threshold-based classification must be performed entirely on-chip, representing an important direction for in-sensor computing.

### 7.4. Array-Level Limitations and Fabrication Challenges

Beyond device- and system-level reliability, the practical realization of in-sensor computing biosensors ultimately requires scalable array-level integration with peripheral CMOS circuits. Therefore, array-level non-idealities and fabrication compatibility must be considered as central bottlenecks for translating memristive biosensing platforms from laboratory-scale demonstrations to manufacturable systems.

As memristive biosensors scale into high-density crossbar arrays, addressing array-level non-idealities such as noise and crosstalk effects becomes imperative. In passive crossbar architectures, sneak path currents significantly degrade computational accuracy and increase energy consumption. To mitigate this without the substantial area overhead of external transistors, recent milestones have demonstrated selector-free, self-rectifying memristive crossbar arrays utilizing transition metal oxides (e.g., HfO_x_, WO_x_) [66,67,68]. By integrating intrinsic diode-like rectification, these architectures effectively suppress leakage paths, achieving remarkable metrics such as kilo-bit array implementations [68] and ultra-high rectification ratios [66,67].

However, these recent self-rectifying crossbar demonstrations do not yet represent complete solutions for practical CMOS foundry-level implementation. Translating these laboratory-scale achievements into practical system-level platforms requires seamless monolithic co-integration with peripheral CMOS circuits on the same substrate. Although BEOL integration within existing CMOS process flows is essential for this purpose, achieving true manufacturing compatibility remains a critical hurdle. In particular, high rectification ratios in state-of-the-art self-rectifying memristors often rely on asymmetric Schottky barriers formed using high-work-function electrode materials such as Pt and Ru. These noble metals are difficult to pattern using standard CMOS dry-etching processes and are therefore frequently implemented through conventional metal lift-off processes, which can limit scalability and foundry-level process compatibility.

Ru integration processes have recently been reported by leading semiconductor foundries. Ru dry-etch processes based on volatile by-products enable subtractive Ru patterning, and a commercial 22ULL embedded ReRAM product has been reported to incorporate an HfO-based resistive layer paired with a Ru layer [69]. In parallel, a subtractive Ru integration process has been demonstrated in R&D test vehicles for CMOS interconnect structures at pitches of 25 nm or below [70]. Although the process currently targets interconnect metallization, it demonstrates the increasing maturity of nanoscale Ru patterning within advanced CMOS process environments. Further validation is required to transfer such process modules to memristive devices while preserving the Ru/oxide interface, switching uniformity, and BEOL process compatibility. Therefore, the development of CMOS-compatible electrode materials, scalable patterning processes, and reliable monolithic co-integration schemes for self-rectifying memristive devices and peripheral circuits remain an important future research direction.

**Figure 12:**
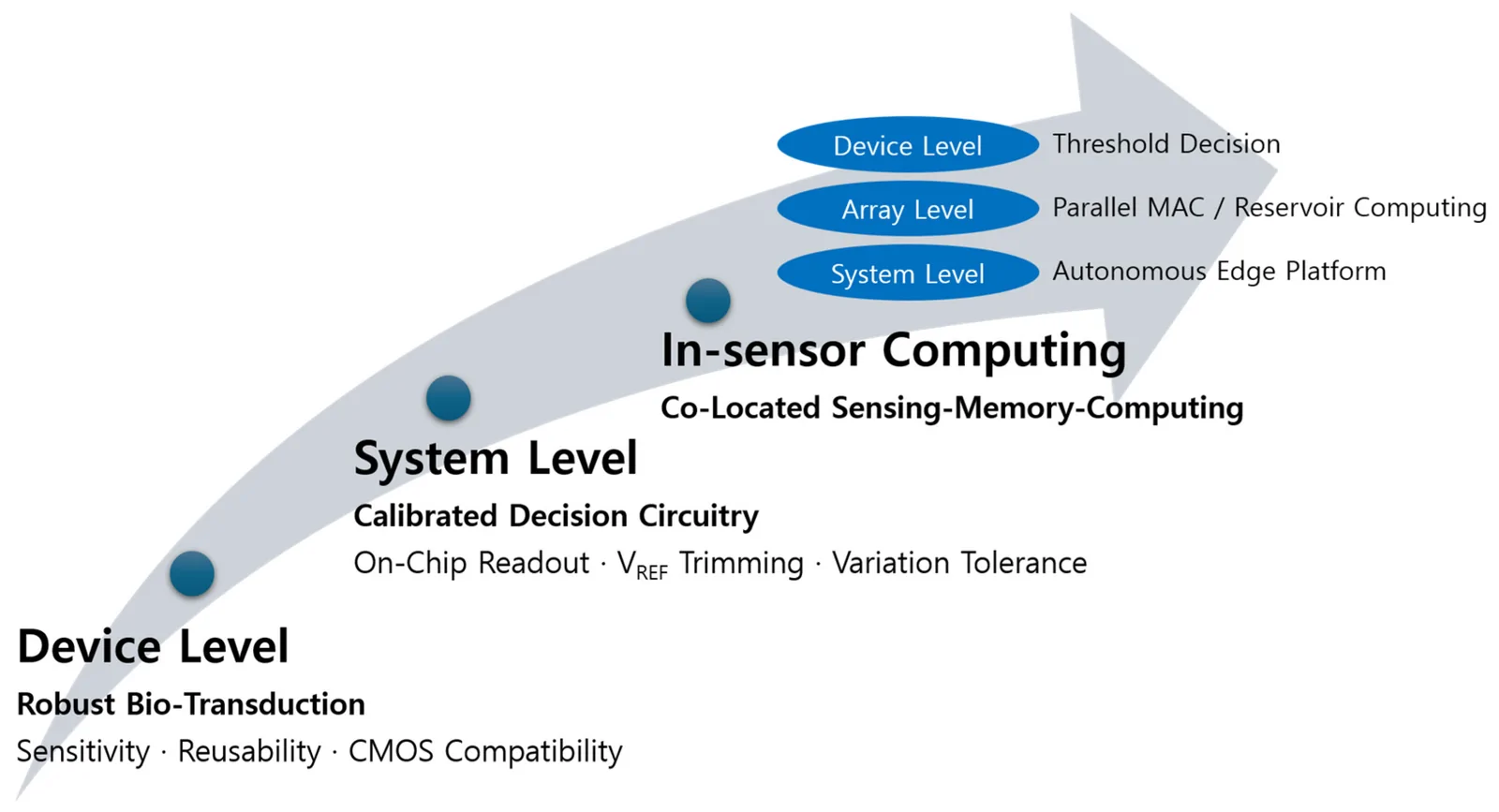
Technological roadmap of memristive-device-based biosensors toward in-sensor computing.

Taken together, the practical realization of memristive-device-based biosensors requires coordinated advances across device, system, and in-sensor computing levels. Figure 12 summarizes this technological progression, highlighting the key requirements for each stage, from robust bio-transduction and calibrated decision circuitry to co-located sensing–memory–computing across device-, array-, and system-level in-sensor computing implementations.

## 8. Conclusions

The integration of memristive devices into electrochemical biosensors represents a significant paradigm shift in diagnostic technologies. As conventional von Neumann architectures encounter fundamental energy and data-transfer bottlenecks, memristors offer a promising solid-state pathway to closely couple biochemical signal transduction with non-volatile memory and local computation.

This review has outlined the architectural evolution of these platforms, driven by the need to overcome inherent physical limitations. Early device-level implementations demonstrated high sensitivity but were limited by irreversible degradation under direct analyte exposure. This limitation motivated the transition toward physically decoupled, indirect sensing architectures. Furthermore, the latency and energy overhead associated with transferring digitized data to external processors in conventional system-level designs have accelerated the development of in-sensor computing architectures.

By co-locating sensing and inference directly at the edge, in-sensor computing has the potential to redefine the form factor, latency, and energy efficiency of POC testing. However, translating these advances from laboratory-scale demonstrations to practical applications requires addressing several key challenges.

As discussed, future progress will rely on achieving CMOS process compatibility and integrating ultra-low-power operation with reliable multi-threshold sensing, thereby enabling quantitative clinical analysis beyond binary detection and supporting wearable, implantable, and ingestible biosensing platforms as a near-term research direction [15,71,72].

## Figures and Tables

**Table 1:** Device-level performance summary of memristive-device-based biosensors and related nanowire sensing platforms.

Ref.	Sensing Route	Target	Device/Readout	LOD	Sensor Sensitivity/ Dynamic Range/ Measurement Time	On-Off Ratio/ Device Reliability	CMOS Compatibility
[32]	Direct FET baseline	HBV-DNA, AFP	SiNW array FET/G	0.1 fM (HBV-DNA), 0.1 fg/mL (AFP)	NR/0.1 fM–100 pM (HBV-DNA), 0.1 fg/mL–1000 pg/mL (AFP)/RT NR	NR/NR	O
[27]	Direct memristive SiNW	anti-PSA antibody	NiSi/SiNW/NiSi/V	NR	NR/NR/NR	NR/NR	X
[28]	Direct memristive aptasensor	PSA	SiNW aptasensor/V	23 aM	NR/aM-pM/ 1 h incubation	NR/NR	X
[29]	Direct liquid bio-memristor	Ebola VP40	Honeycomb SiNW HC-FET/V	6 fM ^†^	NR/6–200 fM/ 30 min incubation, 10 s sweep	NR/NR	X
[35]	Direct oxide/EIS sensing	H_2_O_2_	Cr/BaTiO_x_/TiN RRAM/V	1 nM ^†^	48 mV pH^−1^/ H_2_O_2_ 1–1000 nM, pH 2–10/NR	Up to 2000 / >3 h @ 85 °C, >10^7^ cycles	X
[36]	Direct liquid pH sensing	H^+^ (pH)	Nb–Ti oxide/Pt MIM/R	0.4 pH	0.5 pH^−1^/pH 3–11/NR	Up to ~5/NR	X
[37]	Direct enzymatic urea sensing	Urea	IrOx/GdO_x_/W RRAM/R	0.034 pH	53.2 mV pH^−1^/ pH 6–10/NR	18–12,400/ >10^3^ dc cycles, >10^9^ read cycles	X
[15]	Indirect/ decoupled TS transducer	H^+^ (pH)	TaO_x_/Ta_2_O_5_ memristor + FETs/R	N/A	−57 mV pH^−1^/pH 2–10, TP 10→2/ 0.5–1 min stabilization, 10 × 20 μs pulses	35.2/>30 @ 50 μs, 4–5k pulses; >35 @ 2 μs, 30k pulses	O

^†^ Lowest experimentally tested or reported level, not a statistically calculated LOD. NR, not reported. N/A, not applicable. G, conductance. V, voltage. R, resistance. EIS, electrolyte–insulator–semiconductor. FET, field-effect transistor. HC-FET, honeycomb field-effect transistor. MIM, metal–insulator–metal. RRAM, resistive random-access memory. TP, threshold pH point. TS, threshold sensing.

**Table 2:** System-level implementations of memristive-device-based biosensors and related sensor platforms.

Ref.	System Type	Sensor/Target	Implementation	Analytical Metrics	System Metrics	Power/Time	Key Shift
[38]	Off-chip PCB	Memristive SiNW/PSA	12-ch PCB, MUX, 20-bit ADC, STM32	fM range, LOD NR, RT NR	Automated V_GAP_ extraction, 12 sensors	NR/~40 min → 80 s	Probe-station-free portable readout
[17]	Off-chip smartphone	Au SPE/SLPI	Audio-jack potentiostat, VCO, phone FFT	LOD 1 nM, RT NR	0.3 nA min, 96 dB, 79% harvesting	6.9 mW peak, 2.93 mW idle/NR	Battery-free smartphone POC
[16]	Off-chip microfluidic	Memristive SiNW/antigen	PDMS channel, Al extension readout	fM-order, LOD NR, RT NR	20 µm × 500 µm × 3 mm, 20 µL wash	NR/ 10 min wash, 1 h incubation	Drop-casting → microfluidics
[19]	On-chip neuromorphic	IS-OECT + EC-RAM/sweat Cl^−^, K^+^	Sensor layer, EC-RAM perceptron, LED output	Cl^−^ > 60 mmol L^−1^, LOD NR, RT NR	On-Chip classification, retrainable	Low-voltage operation/NR	External classifier → on-chip learning
[13]	Monolithic 3D benchmark	CNFET + RRAM/ gas-vapor	3D CNFET/RRAM, direct sensor-memory write	LOD N/A, RT ~45 s	1Mbit RRAM, 98.82% yield, 12.5 GB s^−1^	40 μW/ classifier/ 10 μs write	2D → 3D sensing-memory- compute

N/A: not applicable. RT: response time.

**Table 3:** Representative In-Sensor Computing Platforms across Device, Array, and System Levels.

Ref.	Integration Level	Input/ Target Domain	Device/Platform	Integrated Operation	Main Performance	Operational Characteristics/ Implementation Status
[15]	Device	pH/electrochemical	TaO_x_/Ta_2_O_5_ RRAM + FETs	Threshold-sensing transducer	−57 mV pH^−1^, pH 2–10, r = 0.981	20 μs × 10 pulses, 12.4× power reduction/prototype
[20]		Vision/optical image	a-Si PIN PD + poly-Si TFT	Light-to-spike + 3 × 3 convolution	0.1–12 mW cm^−2^, R^2^ > 0.99, 95% accuracy	200 Hz–1.2 kHz/ device demo + SNN simulation
[21]		Tactile/breath	TENG + biristor neuron	Pressure-to-spike encoding	~3 kPa, MNIST 85.8%, breath demo	0.98 nJ/spike, self-powered/ device + breath HW demo
[45]	Array	Biomedical sequence	Pt/gradual TiO_x_/ Ti 20 × 20 array	Volatile temporal kernel	AMP prediction 84.11%, On-Off Ratio > 2000	10–35 μA, >40 μs/ 20 × 20 array
[48]		Temporal data/ MNIST/MGTS	W/HfO_2_/TiN 2M1C kernel	RC-delay dual mapping	MNIST 94.3%, MGTS NRMSE 0.04	5 V, 50 μs/10 × 1 array
[40]		Vision/ static image	Phototransistor + GST PCM	PCM-weighted pixel MAC	Gaussian blur, Hough line detection	NR/toy pixel + PCM chip
[49]		ECG/vision	Ag/ZnWO_4−x_/ WO_3−x_/ITO OEM	Memristor reservoir	ECG 96.84%, face 92.73%	NR/ dataset-based RC demo
[50]		EEG + snoring/sleep	Ag/MoS_2_ QDs/Ag fiber memristor	Fiber RC + CNN	Snoring 94.8%, sleep 95.4%, fusion 93.5%	±1 V, sub-nA, pW-level power/ textile integrated RC demo
[51]		PPG/ECG/ accelerometer	DYNAP-SE mixed-signal processor	LIF + decoder/NSM	HR RRMSE ~1 bpm	74.7 μW total/ HW-validated
[22]		EMG/gesture	Stretchable ISOECT array	EMG sensing + RC	Gesture ~90%, >50% strain, Gm ~1 mS	Sensor 0.035 nJ/op, RC < 0.56 nJ/op/ wearable HW demo
[53]		Vision/display	GaN/(In,Ga)N micro-LED	Recognition + display	R ~2.2 mA W^−1^, >88%	41 ms rise, 37 ms fall, 4.5% energy saving/ device demo + SNN simulation
[54]		Video/visual scene	32 × 32 selector-less TiO_x_ 1R array	Analog MAC + self-calibration	PSNR 30.49 dB, SSIM 0.81	~0.7 fps/ 32 × 32 array HW demo + simulation
[55]	System	COVID-19 antibody	Vertical meshed electrochemical chip	SERM duplex readout	LOD 8/83.1 ng mL^−1^, 100%	NR/portable serum demo
[57]		Epidermal flux	Non-contact EFS wearable	Wireless chamber + model	TEWL r = 0.98, mass loss r = 0.99	≥1 day operation, <10 ms valve closing/ wireless wearable demo
[23]		Gesture/gait	AlN MRR + Si MZI PIC	Photonic FE + PNN	Gesture 96.77%, gait 98.31%	<0.34 pJ, <10 ns/ PIC + wearable TENG demo

RRAM: resistive random-access memory; FET: field-effect transistor; PD: photodiode; TFT: thin-film transistor; SNN: spiking neural network; TENG: triboelectric nanogenerator; MNIST: Modified National Institute of Standards and Technology; AMP: antimicrobial peptide; RC: reservoir computing; MGTS: Mackey–Glass time series; NRMSE: normalized root-mean-square error; 2M1C: two-memristor–one-capacitor; GST: Ge_2_Sb_2_Te_5_; PCM: phase-change memory; MAC: multiply-and-accumulate; ECG: electrocardiogram; OEM: optoelectronic memristor; EEG: electroencephalogram; QDs: quantum dots; CNN: convolutional neural network; PPG: photoplethysmography; DYNAP-SE: Dynamic Neuromorphic Asynchronous Processor; HR: heart rate; RRMSE: relative root-mean-square error; LIF: leaky integrate-and-fire; NSM: neural state machine; EMG: electromyography; ISOECT: intrinsically stretchable organic electrochemical transistor; PSNR: peak signal-to-noise ratio; SSIM: structural similarity index measure; SERM: single-response electrochemical multiplexing; LOD: limit of detection; EFS: epidermal flux sensor; TEWL: transepidermal water loss; MRR: microring resonator; MZI: Mach–Zehnder interferometer; PIC: photonic integrated circuit; FE: feature extraction; PNN: photonic neural network; HW: hardware.

## Data Availability

No new data were created or analyzed in this study. Data sharing is not applicable to this article.

## Abbreviations

The following abbreviations are used in this manuscript:

ADC	Analog-to-Digital Converter
AFP	Alpha-Fetoprotein
AI	Artificial Intelligence
ANN	Artificial Neural Networks
BEOL	Back-End-Of-Line
CDW	Charge Density Waves
CMOS	Complementary Metal-Oxide-Semiconductor
CNFET	Carbon-Nanotube Field-Effect Transistor
DAC	Digital-to-Analog Converter
DSP	Digital Signal Processor
ECG	Electrocardiogram
EC-RAM	Electrochemical Random-Access Memory
EEG	Electroencephalogram
EMG	Electromyography
FET	Field-Effect Transistor
HBV	Hepatitis B Virus
HRS	High Resistance State
IUPAC	International Union of Pure and Applied Chemistry
I-V	Current-Voltage
ISOECT	Intrinsically Stretchable Organic Electrochemical Transistor
IS-OECT	Ion-Selective Organic Electrochemical Transistor
LED	Light-Emitting Diode
LOD	Limit of Detection
LRS	Low Resistance State
MAC	Multiply-and-Accumulate
MCU	Microcontroller Unit
MFC	Microbial Fuel Cell
MZI	Mach-Zehnder Interferometer
NIL	Nanoimprint lithography
OECT	Organic Electrochemical Transistor
OEM	Optoelectronic Memristors
PBS	Phosphate-Buffered Saline
PCB	Printed Circuit Board
PCM	Phase-Change Memory
PEG	Polyethylene Glycol
PIC	Photonic Integrated Circuit
PIN	Positive-Intrinsic-Negative
POC	Point-of-Care
PSA	Prostate Specific Antigen
PSNR	Peak Signal-to-Noise Ratio
RC	Reservoir Computing
ROIC	Readout Integrated Circuit
ROS	Reactive Oxygen Species
RRAM	Resistive Random-Access Memory
SEM	Scanning Electron Microscope
SERM	Single-Response Multiplexing
SiNW	Silicon Nanowire
SLPI	Secretory Leukocyte Protease Inhibitor
SNN	Spiking Neural Network
TENG	Triboelectric Nanogenerator
TFT	Thin-Film Transistor
TS	Threshold Sensing
VCO	Voltage-Controlled Oscillator
VOC	Volatile Organic Compounds
1T1R	One-Transistor One-Resistor
2M1C	Two-Memristor One-Capacitor
1D	One-Dimensional
2D	Two-Dimensional
3D	Three-Dimensional
